# Arctic Ocean annual high in ${{\boldsymbol{p}}}_{{{\bf{CO}}}_{{\bf{2}}}}$ could shift from winter to summer

**DOI:** 10.1038/s41586-022-05205-y

**Published:** 2022-10-05

**Authors:** James C. Orr, Lester Kwiatkowski, Hans-Otto Pörtner

**Affiliations:** 1 LSCE-IPSL, Laboratoire des Science du Climat et de l’Environnement, CEA-CNRS-UVSQ, CEA Saclay, Gif-sur-Yvette, France; 2 LOCEAN-IPSL, Laboratoire d’Océanographie et du Climat: Expérimentations et Approches Numériques, Sorbonne Université-CNRS-IRD-MNHN, Paris, France; 3 Integrative Ecophysiology, Alfred Wegener Institute, Helmholtz Centre for Polar and Marine Research, Bremerhaven, Germany

**Keywords:** Carbon cycle, Marine chemistry

## Abstract

Long-term stress on marine organisms from ocean acidification will differ between seasons. As atmospheric carbon dioxide (CO_2_) increases, so do seasonal variations of ocean CO_2_ partial pressure (${p}_{{{\rm{CO}}}_{2}}$), causing summer and winter long-term trends to diverge^1–5^. Trends may be further influenced by an unexplored factor—changes in the seasonal timing of ${p}_{{{\rm{CO}}}_{2}}$. In Arctic Ocean surface waters, the observed timing is typified by a winter high and summer low^6^ because biological effects dominate thermal effects. Here we show that 27 Earth system models simulate similar timing under historical forcing but generally project that the summer low, relative to the annual mean, eventually becomes a high across much of the Arctic Ocean under mid-to-high-level CO_2_ emissions scenarios. Often the greater increase in summer ${p}_{{{\rm{CO}}}_{2}}$, although gradual, abruptly inverses the chronological order of the annual high and low, a phenomenon not previously seen in climate-related variables. The main cause is the large summer sea surface warming^7^ from earlier retreat of seasonal sea ice^8^. Warming and changes in other drivers enhance this century’s increase in extreme summer ${p}_{{{\rm{CO}}}_{2}}$ by 29 ± 9 per cent compared with no change in driver seasonalities. Thus the timing change worsens summer ocean acidification, which in turn may lower the tolerance of endemic marine organisms to increasing summer temperatures.

## Abstract (web summary)

Simulations suggest that the partial pressure of carbon dioxide in the Arctic Ocean will shift from a winter to a summer maximum owing to enhanced summer sea surface warming from earlier sea-ice retreat.

## Main

More than a decade ago, it was recognized that the ongoing increase in atmospheric carbon dioxide (CO_2_) would affect not only the annual mean state of ocean CO_2_ system variables but also their seasonal variations. Theory suggests that seasonal variations in ocean pH and CO_2_ partial pressure (${p}_{{{\rm{CO}}}_{2}}$) should increase^9^ as more CO_2_ invades the ocean and its buffer capacity is reduced, consistent with earlier model and mesocosm studies^10,11^. Subsequent observations and model projections confirm the proposed increase in the seasonal amplitude of ${p}_{{{\rm{CO}}}_{2}}$ across the ocean, although the Arctic Ocean was either excluded from the analysis^1,4^ or included while presuming that effects from physical climate change were negligible^2^. For pH, models project that generally the amplitude of seasonal variations will actually decline because its changes are relative, not absolute changes in hydrogen ion concentration [H^+^] owing to the logarithmic transformation; conversely, seasonal variations in [H^+^] increase nearly linearly with those for ${p}_{{{\rm{CO}}}_{2}}$ (refs. ^3,5^).

Here we have assessed monthly variations and their potential future changes in timing for surface ocean ${p}_{{{\rm{CO}}}_{2}}$ and related ocean CO_2_ system variables in the Arctic Ocean using a suite of 27 Earth system models (ESMs) that participated in the two most recent phases of the Coupled Model Intercomparison Project (CMIP5 and CMIP6; Supplementary Table 1). Driving mechanisms were assessed with Taylor-series expansions and idealized experiments from CMIP5 to distinguish the direct chemical consequences attributable to the increase in atmospheric CO_2_ from the indirect consequences of physical climate change (Methods). Here these tools are used to focus on the timing of the annual cycle and its future change in regards to chemical and biological impacts.

## Timing of annual cycle

For the modern ocean, the timing of the annual cycle of ${p}_{{{\rm{CO}}}_{2}}$ and related variables is generally well understood. In the subtropics, where the annual cycle of ${p}_{{{\rm{CO}}}_{2}}$ is dominated by temperature-driven variations, observations indicate that ${p}_{{{\rm{CO}}}_{2}}$ levels are consistently higher in summer and lower in winter, whereas subpolar regions have the opposite pattern because non-thermal effects dominate^12,13^. Similar seasonal patterns and driving mechanisms are simulated by the CMIP5 models under modern forcing^3,4^ except in the Southern Ocean where models struggle^14,15^. However, modelled seasonal variations in ${p}_{{{\rm{CO}}}_{2}}$ have not been evaluated in the Arctic Ocean owing to the sparsity of seasonal observations. To fill this observational gap, we exploited a recent neural-network-derived, high-resolution, observation-based product^6^ that includes the non-coastal Arctic Ocean. It indicates that surface-water ${p}_{{{\rm{CO}}}_{2}}$ is lower in summer than in winter when averaged across the Arctic, suggesting that non-thermal effects dominate as in the subarctic^12,13^ (Supplementary Fig. 1). The models generally show the same tendency for present-day monthly variations of the basin-wide mean, a major component of overall seasonal variability (Supplementary Fig. 2) that correlates strongly with the observation-based variations (Supplementary Fig. 3). This consistency improves confidence in the future projections.

**Fig. 1:**
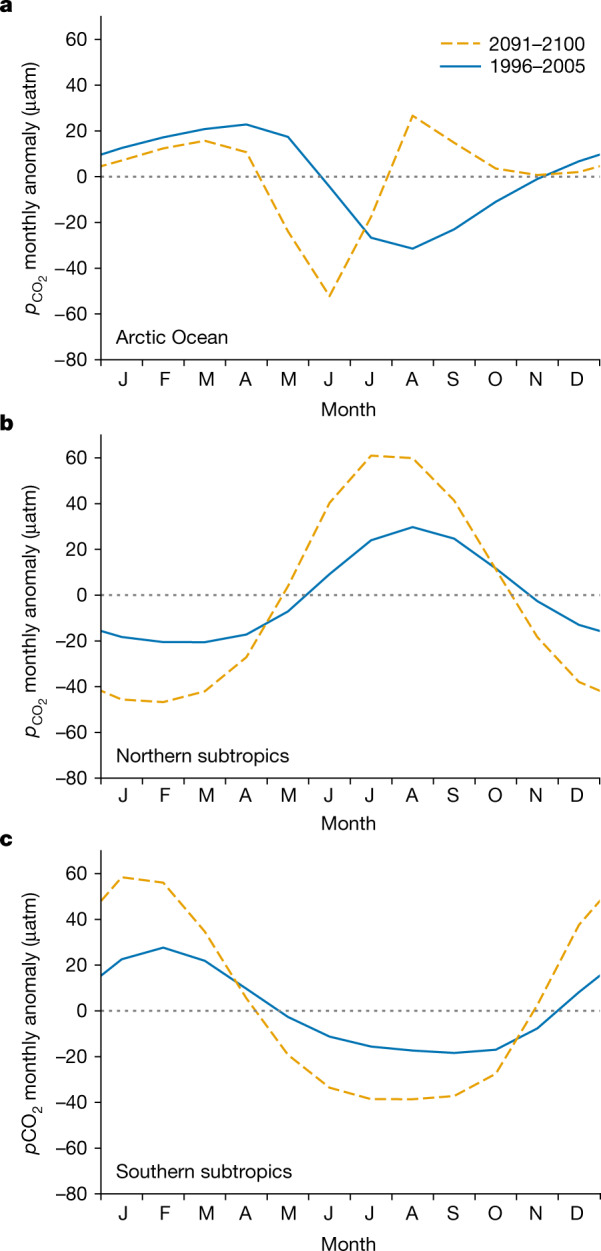
Projected seasonal timing of ${{\boldsymbol{p}}}_{{{\bf{CO}}}_{{\bf{2}}}}^{{\prime} }$ changes little except in the Arctic Ocean. **a**–**c**, Curves represent the CMIP5 model mean for 1996–2005 (historical) and 2091–2100 (RCP8.5) detrended decadal climatologies averaged over the Arctic Ocean (**a**), the northern subtropics (**b**) and the southern subtropics (**c**).

**Fig. 2:**
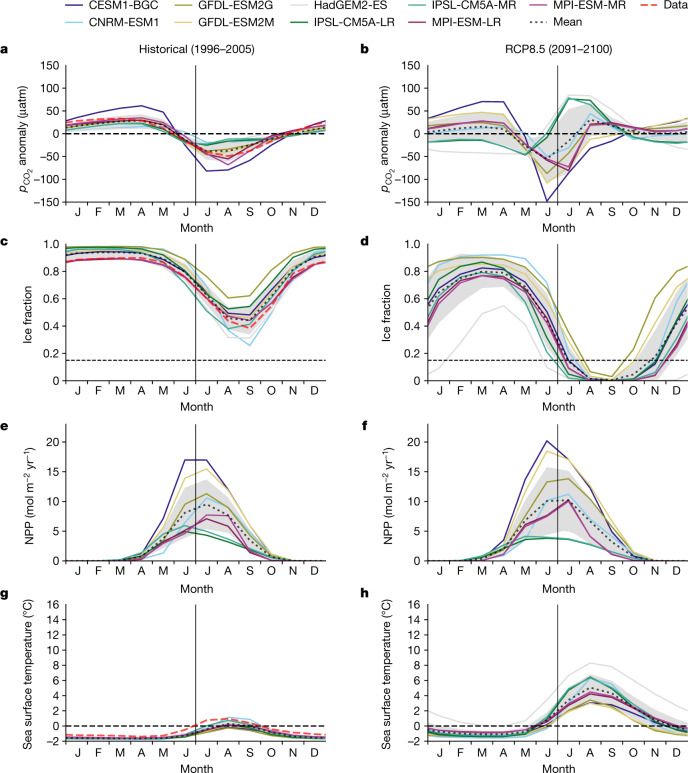
Splitting and inversion of summer low tends to occur for ${{\boldsymbol{p}}}_{{{\bf{CO}}}_{{\bf{2}}}}^{{\prime} }$ but not its drivers. **a**–**h**, Arctic domain averages for decadal climatologies of 1996–2005 (**a**,**c**,**e**,**g**) and 2091–2100 (**b**,**d**,**f**,**h**) are shown for nine CMIP5 models (historical and RCP8.5) for surface ocean ${p}_{{{\rm{CO}}}_{2}}^{{\prime} }$ (**a**,**b**), fractional ice concentration (**c**,**d**), NPP (**e**,**f**) and surface ocean temperature (**g**,**h**). The line colours represent individual models, the black dots represent the model mean and the shaded region is the uncertainty (±1 s.d., *n* = 9). The red dashes are for modern observational estimates (gridded data products) for ${p}_{{{\rm{CO}}}_{2}}$, ice fraction and sea surface temperature (Methods). Extended Data Fig. 1 shows analogous results from CMIP6 (SSP5-8.5). Models fall into three groups for simulated ${p}_{{{\rm{CO}}}_{2}}^{{\prime} }$ in 2091–2100 but may share a common evolution pathway (Fig. 3).

**Extended Data Fig. 1:**
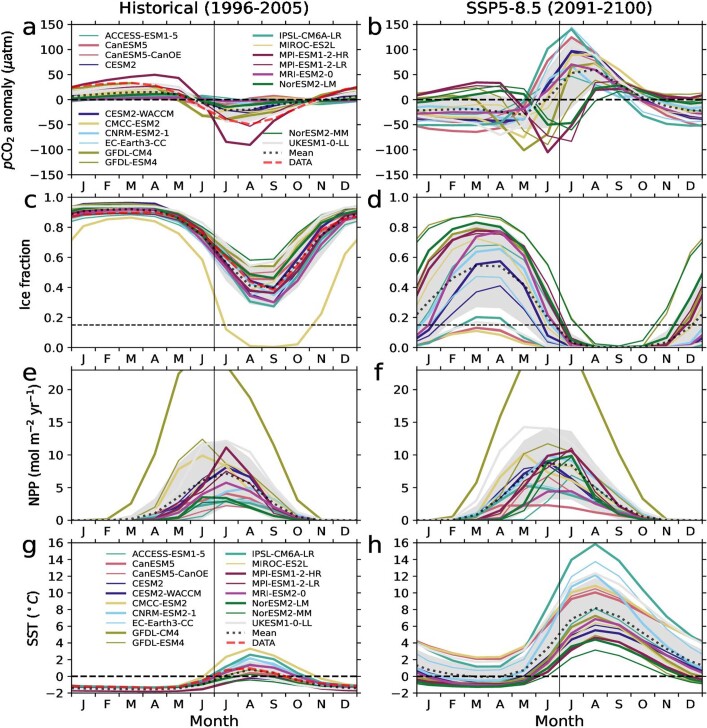
CMIP6 also reveals splitting and inversion of the summer low for ${{\boldsymbol{p}}}_{{{\bf{CO}}}_{{\bf{2}}}}^{{\prime} }$ but not its drivers. Monthly variations averaged over the Arctic domain for decadal climatologies of 1996–2005 (**a, c, e, g**) and 2091–2100 (**b, d, f, h**) are shown for 18 CMIP6 models (historical and SSP5-8.5) for **a, b**, surface ocean ${p}_{{{\rm{CO}}}_{2}}^{{\prime} }$, **c-d**, fractional ice concentration, **e, f**, NPP, and **g, h**, surface ocean *T*. Line colours represent individual models and black dots the model mean, while the shaded region is the uncertainty (±1 s.d., n = 18). Red dashes are for modern observational estimates (gridded data products) for ${p}_{{{\rm{CO}}}_{2}}$, ice fraction, and SST. Fig. 2 shows analogous results from CMIP5 (RCP8.5).

Under high-end emissions, the models project that by 2100, the thermally driven, summer maximum in ${p}_{{{\rm{CO}}}_{2}}$ in the low latitudes occurs about a month earlier, as does the biologically driven, summer minimum in the high latitudes (Fig. 1). However, in the Arctic, today’s broad summer minimum in the monthly anomaly of ${p}_{{{\rm{CO}}}_{2}}$, that is, relative to the annual mean and denoted as ${p}_{{{\rm{CO}}}_{2}}^{{\prime} }$, tends to narrow in the future projections of all models, often splitting into a spring–summer minimum and a summer maximum (Fig. 2 and Extended Data Fig. 1). That projected earlier minimum in ${p}_{{{\rm{CO}}}_{2}}^{{\prime} }$ usually occurs along with an earlier peak in net primary production (NPP), whereas the summer ${p}_{{{\rm{CO}}}_{2}}^{{\prime} }$ maximum occurs along with a sharp increase in summer sea surface temperature (*T*). Both appear to be tied to earlier retreat of seasonal sea-ice cover. These projected tendencies are consistent with observations in some Arctic regions that are already experiencing earlier reductions in sea-ice cover, where phytoplankton blooms occur earlier in the year^16^ and there is greater surface warming^8^. Observed summer ${p}_{{{\rm{CO}}}_{2}}$ is higher in low-ice years^17^, has grown over recent decades as sea ice retreats^18^ and is particularly high under exceptional warming^19–21^.

**Extended Data Fig. 2:**
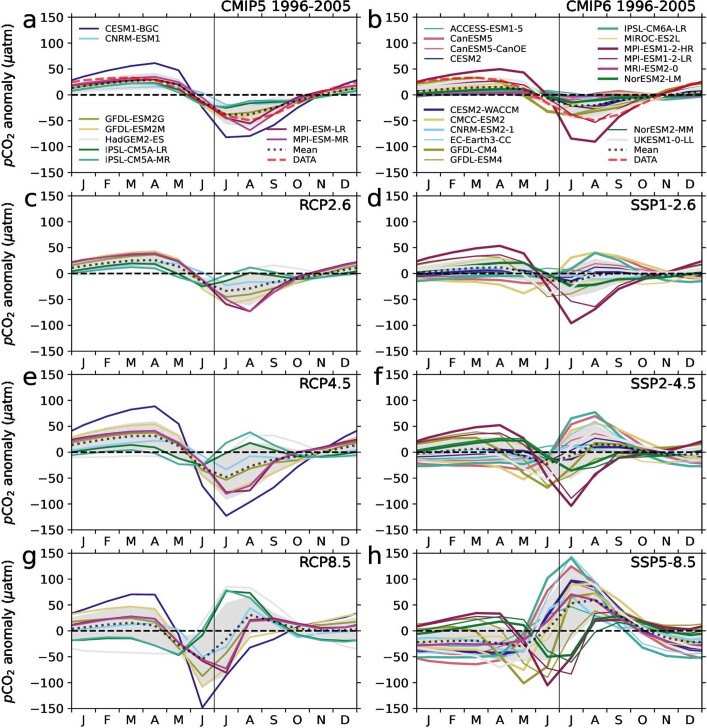
Summer ${{\boldsymbol{p}}}_{{{\bf{CO}}}_{{\bf{2}}}}^{{\prime} }$ turns positive in most models under mid-range to high-end emissions. All panels show climatological averages of surface ocean ${p}_{{{\rm{CO}}}_{2}}^{{\prime} }$ averaged over the Arctic domain. Columns are for CMIP5 (**a, c, e, g**) and CMIP6 (**b, d, f, h**). Rows are for **a, b**, 1996– 2005 from the historical experiments and for 2091–2100 from the RCP and SSP scenarios at **c, d**, 2.6 **e, f**, 4.5, and **g, h**, 8.5 W m^−2^ (see Methods). Line colours represent individual models and black dots the multimodel mean, while the shaded region is the uncertainty (±1 s.d., n = 9 for CMIP5, n = 18 for CMIP6).

The projected sign reversal in the summer extreme of ${p}_{{{\rm{CO}}}_{2}}^{{\prime} }$ occurs in most models under high-end emissions, in many models under mid-range emissions and in some models under low-end emissions (Extended Data Fig. 2).

**Fig. 3:**
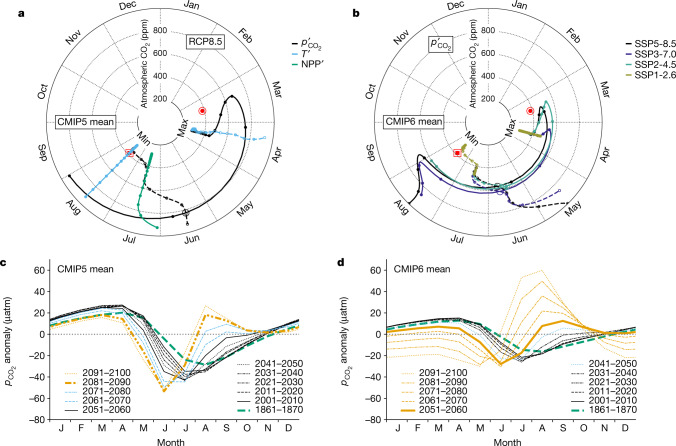
Seasonal timing of ${{\boldsymbol{p}}}_{{{\bf{CO}}}_{{\bf{2}}}}^{{\prime} }$ evolves gradually in three stages and the annual high and low eventually cross. **a**–**d**, Evolution of CMIP means averaged over the Arctic domain for annual highs and lows (**a**,**b**), where the angle is the month and the radius is the atmospheric CO_2_ level, and the full seasonal cycle (**c**,**d**). **a**, CMIP5 results (historical + RCP8.5) for ${p}_{{{\rm{CO}}}_{2}}^{{\prime} }$ (black), sea surface temperature (light blue) and NPP (green). **b**, CMIP6 ${p}_{{{\rm{CO}}}_{2}}^{{\prime} }$ in four SSP scenarios. Shown are annual highs (solid) and lows (dashed) and ${p}_{{{\rm{CO}}}_{2}}^{{\prime} }$ crossover points (large open circles). The low for NPP is indistinct (Fig. 2) and not shown. Encircled points are the ${p}_{{{\rm{CO}}}_{2}}$ data product (Methods). Small circles mark the end of decades (2100, 2090, 2080 and so on) but end in 2090 for SSP5-8.5. **c**,**d**, Evolution of the full seasonal cycle (decadal averages) of ${p}_{{{\rm{CO}}}_{2}}^{{\prime} }$ in CMIP5 (**c**) and CMIP6 (**d**) means occurs in three stages: (1) no maximum in summer (black), (2) positive secondary maximum in summer (light blue) and (3) annual maximum in summer (orange). The thick orange line indicates the first decade reaching stage 3; line patterns distinguish different decades. This evolution pathway is the rule among models but some do not make the full journey (Supplementary Figs. 4 and 5).

The evolution in timing of the annual low and high in Arctic Ocean ${p}_{{{\rm{CO}}}_{2}}^{{\prime} }$ differs from other variables such as NPP and *T*, which exhibit little or no timing change (Fig. 3a). Throughout the industrial era until present, the annual high in ${p}_{{{\rm{CO}}}_{2}}^{{\prime} }$ occurs in April for the CMIP5 mean averaged over the Arctic domain. But as atmospheric CO_2_ continues to increase, the timing of that annual high first retreats to March and then advances rapidly, finishing in September at the end of the century (936 ppm under Representative Concentration Pathway 8.5 (RCP8.5)). Simultaneously, the timing of the annual low retreats from August to June, crossing over the advancing annual high at 827 ppm. The same abrupt transition is found for the CMIP6 model mean but crossover occurs at 571 ppm, and the advance of the timing of the annual high to September reverses itself at about 700 ppm and then recedes to August (Fig. 3b). Although the crossover CO_2_ level differs by 256 ppm between CMIP5 and CMIP6 means, and by more among models as shown below, it is remarkably consistent across scenarios for a given model. Despite the abrupt transition in the annual maximum ${p}_{{{\rm{CO}}}_{2}}^{{\prime} }$ from winter to summer, the increase in summer ${p}_{{{\rm{CO}}}_{2}}^{{\prime} }$ is a gradual evolution (Fig. 3c,d), the range of which seems to contain the different model behaviours seen in 2091–2100 (Fig. 2 and Extended Data Fig. 1).

**Extended Data Fig. 3:**
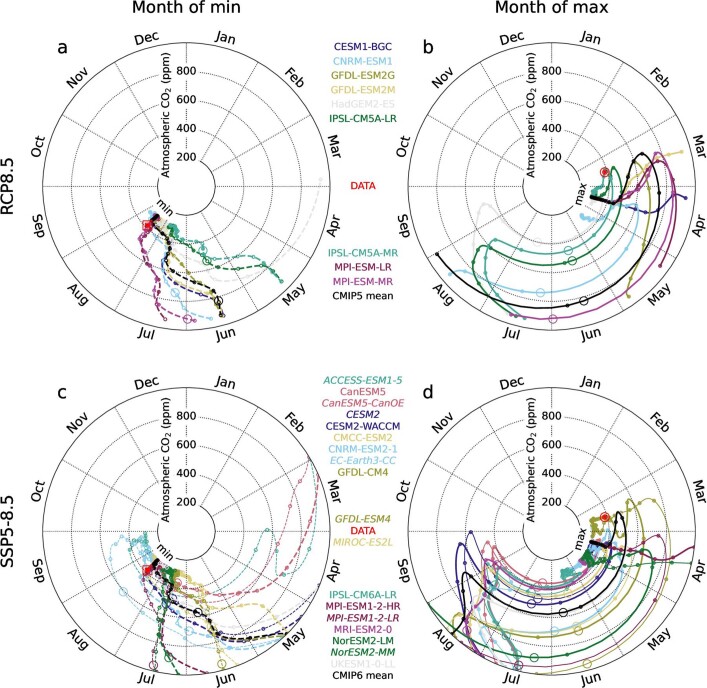
Most models exhibit crossover but at varying levels of atmospheric CO_2_. Evolution of timing of annual highs and lows in ${p}_{{{\rm{CO}}}_{2}}$ averaged over the Arctic domain, combining historical and high-end scenarios. Columns are for the **a, c**, annual low and **b,d**, annual high; rows are for **a, b**, CMIP5 RCP8.5 and **c, d**, CMIP6 SSP5-8.5 models. In **c**, IPSL-CM6A-LR and MRI-ESM2-0 are not shown due to noise from averaging over a broad minimum spanning January (Dec-Mar). Plot characteristics are as in Fig. 3. Italic model labels indicate thin lines. Red points are the observation-based product for ${p}_{{{\rm{CO}}}_{2}}$. Small circles mark the end of decades (2100, 2090, 2080 ...), but 2090 is reached in SSP5-8.5 at the 1000-ppm limit of the plot domain.

**Extended Data Fig. 4:**
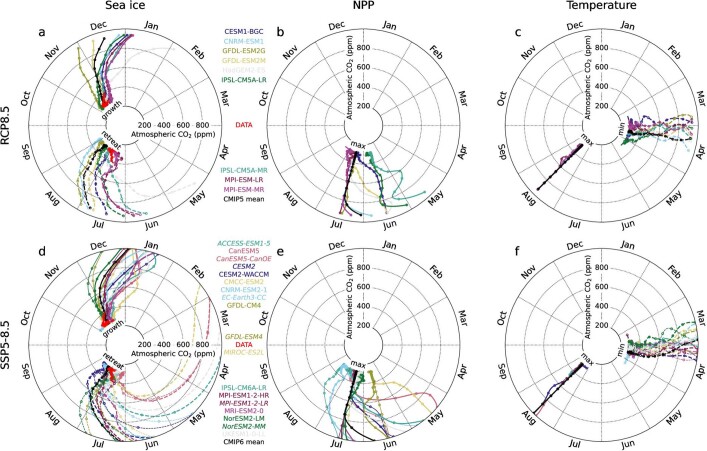
Drivers of ${{\boldsymbol{p}}}_{{{\bf{CO}}}_{{\bf{2}}}}$ do not exhibit crossover in any CMIP model despite large changes in sea ice. Seasonal evolution of ${p}_{{{\rm{CO}}}_{2}}$-related drivers in the CMIP models for their timing of **a, d**, sea-ice retreat and regrowth (see Methods), **b, e**, annual high of net primary production, and **c, f**, annual low and high of temperature are shown for both **a–c**, CMIP5 RCP8.5 and **d–f**, CMIP6 SSP5-8.5. Curves indicate Arctic domain averages for each model, while red points are for the observation-based product of sea-ice concentration (Methods). Plot characteristics are as in Fig. 3 and Extended Data Fig. 3.

Crossover for ${p}_{{{\rm{CO}}}_{2}}^{{\prime} }$ occurs in most models under mid- and high-level emissions (Extended Data Fig. 3) but never for related drivers (Extended Data Fig. 4). For CMIP5, five of nine models reach ${p}_{{{\rm{CO}}}_{2}}^{{\prime} }$ crossover, in June or July at atmospheric CO_2_ levels that vary from 400 ppm to 922 ppm. Two CMIP5 models do not reach crossover but the timing of their annual highs and lows converge. Those seven models all have a new maximum in summer, with four also retaining the spring maximum; the two remaining CMIP5 models fail to converge and have only a spring maximum (Fig. 2). For CMIP6, all models reach positive ${p}_{{{\rm{CO}}}_{2}}^{{\prime} }$ in summer, with 16 crossing over at atmospheric CO_2_ levels between 365 ppm and 959 ppm. Models with earlier crossover generally have higher equilibrium climate sensitivity (the global-mean change in surface atmospheric temperature eventually reached after atmospheric CO_2_ is doubled) and higher area-averaged, summer maximum sea surface temperature in 2091–2100 (Supplementary Table 2).

**Fig. 4:**
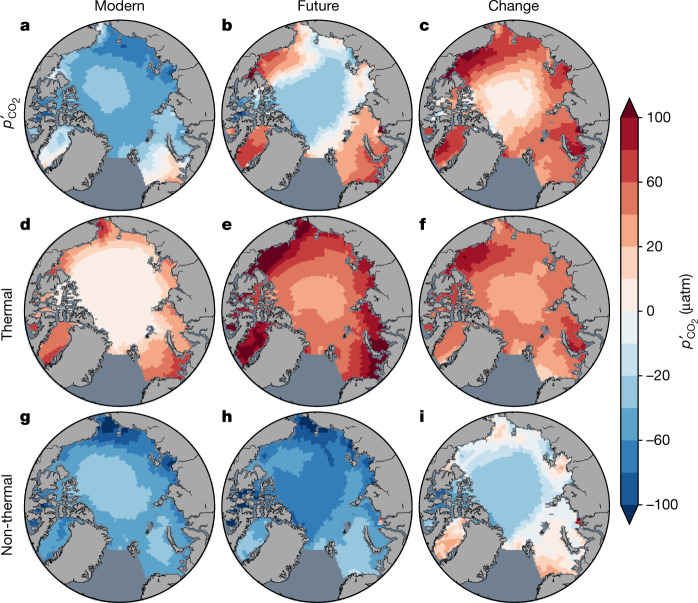
Future summer ${{\boldsymbol{p}}}_{{{\bf{CO}}}_{{\bf{2}}}}^{{\prime} }$ is dominated by warming, particularly in shelf seas. **a**–**i**, Arctic maps of the summer anomalies of total ${p}_{{{\rm{CO}}}_{2}}$ (**a**–**c**), the thermal component (**d**–**f**) and the non-thermal component (**g**–**i**) are shown for the CMIP5 mean (RCP8.5) as decadal averages for 2006–2015 (modern; **a**,**d**,**g**), 2091–2100 (future; **b**,**e**,**h**) and their difference (**c**,**f**,**i**). The summer anomaly is the average of the monthly anomalies over the three summer months (June, August, September). The components are from the Taylor expansion. The non-thermal component can be further decomposed into its various contributions, as discussed later, showing for instance that its reduction on the shelves is mostly from reduced influence of *C*_T_.

The tendency for ${p}_{{{\rm{CO}}}_{2}}^{{\prime} }$ to reverse sign in summer during this century is particularly pronounced in the Arctic shelf seas (Fig. 4). Despite their diversity, most CMIP5 models exhibit this sign reversal over most of the shelf seas, which make up about half of the Arctic Ocean’s surface area (Supplementary Fig. 6). For example, in the Barents Sea, all CMIP models assessed already simulate an annual high in regional mean ${p}_{{{\rm{CO}}}_{2}}^{{\prime} }$ in summer or they project crossover by 2100 under high-end emissions; the same holds in the Kara and Chukchi seas barring one CMIP5 model (Supplementary Figs. 7 and 8). Models also project enhanced summer warming across the Arctic, with generally more in the shelf seas (Supplementary Fig. 9) where sea ice recedes earlier. Positive ${p}_{{{\rm{CO}}}_{2}}^{{\prime} }$ occurs in summer with enhanced warming in regions where the date of sea-ice retreat occurs before 1 August, referred to as the late-summer transition, after which the declining net air-to-sea heat flux fails to warm surface waters^8^ (Supplementary Fig. 10).

Yet warming from sea-ice retreat does not act alone on ${p}_{{{\rm{CO}}}_{2}}^{{\prime} }$. Generally opposed in sign is the non-thermal contribution, which varies among models, for example, owing to different biogeochemical components. It is the balance of the non-thermal contribution with the gradually increasing thermal contribution that determines when summer ${p}_{{{\rm{CO}}}_{2}}^{{\prime} }$ becomes positive and when it surpasses the winter maximum, both abrupt transitions. Models differ in ${p}_{{{\rm{CO}}}_{2}}^{{\prime} }$ because non-thermal as well as thermal contributions differ, as suggested by their large differences in *T*′ and NPP^5^ (Fig. 2 and Extended Data Fig. 1). Although modelled NPP varies greatly and is imperfect, for example, not capturing observation-based estimates of regional differences^22^ and recent changes in phenology^23,24^, and observations cannot tell us whether NPP will continue to increase^25^ or decline^26^ during this century, the use of many diverse models lends confidence that they would encompass the real ocean response. Weighing these contributions requires a quantitative framework.

## Quantifying drivers

**Fig. 5:**
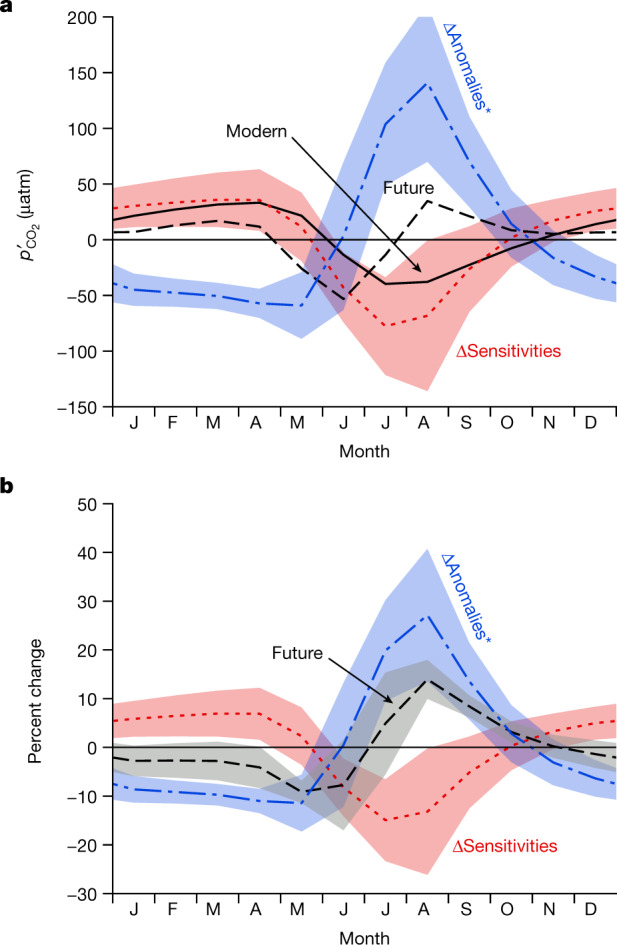
Changes in driver anomalies enhance extreme summer ${{\boldsymbol{p}}}_{{{\bf{CO}}}_{{\bf{2}}}}^{{\prime} }$ by 29 ± 9%. **a**,**b**, Modern and future monthly anomalies of ${p}_{{{\rm{CO}}}_{2}}$ from altered sensitivities and driver anomalies are shown in terms of absolute changes (**a**) and percent changes relative to the modern state (**b**). The black lines show the CMIP5 mean in 2006–2015 (modern; solid) and 2091–2100 (future; dashed) in terms of monthly anomalies relative to the annual mean; the coloured lines indicate the changes between those decades from changing sensitivities (red dots) and changing driver anomalies including the synergy term (blue dot-dash). Shading indicates model agreement (±1 s.d., *n* = 9) but is omitted for the two black curves in **a**, for which it was already shown in Fig. 2.

**Extended Data Fig. 5:**
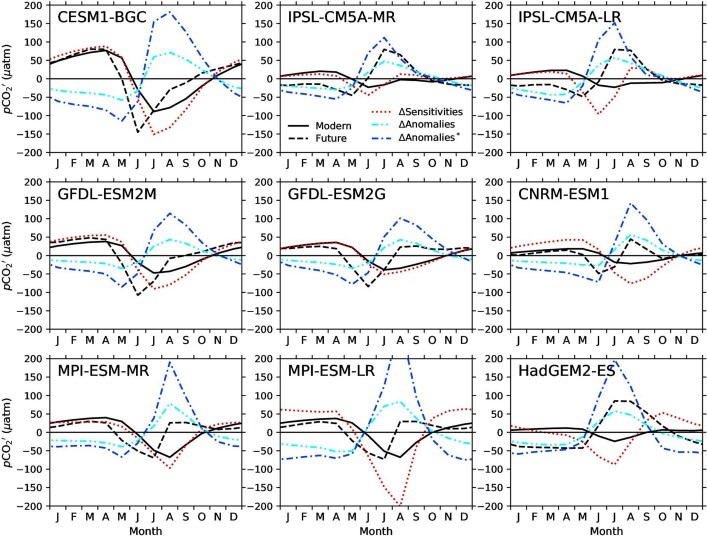
Summer ${{\boldsymbol{p}}}_{{{\bf{CO}}}_{{\bf{2}}}}^{{\prime} }$ increases because effects from changing driver anomalies outweigh those from changing sensitivities. Climatologies are shown for each CMIP5 model under RCP8.5 for ${p}_{{{\rm{CO}}}_{2}}^{{\prime} }$ in 2006–2015 (modern, black solid) and 2091–2100 (future, black dashes) as well as contributions to the change between the two periods, assessed with the climate–CO_2_ Taylor separation, from changing sensitivities (red dots) and changing driver anomalies alone (cyan dot-dot-dash). The latter is also shown combined with the synergy term, i.e. its amplification from the change in sensitivities (dark blue dash-dots). The dashed black curve for ${p}_{{{\rm{CO}}}_{2}}^{{\prime} }$ in 2091–2100 is the sum of the black solid, red dotted, and dark blue dash-dotted curves (equation 3).

A first framework, three idealized experiments run in two CMIP5 models, suggests that the ${p}_{{{\rm{CO}}}_{2}}^{{\prime} }$ sign reversal is steered by indirect effects of physical climate change and amplified by direct effects of higher atmospheric CO_2_ on ocean chemistry (Supplementary Section 1). A second framework was used to assess contributions in the non-idealized experiments made with all CMIP5 models. Contributions to variations in ${p}_{{{\rm{CO}}}_{2}}$ are often deconvolved term by term with a Taylor expansion^12^

1 $${p}_{{{\rm{CO}}}_{2}}^{{\prime} }\approx \frac{\partial \,{p}_{{{\rm{CO}}}_{2}}}{\partial T\,}{T}^{{\prime} }+\frac{\partial \,{p}_{{{\rm{CO}}}_{2}}}{\partial S\,}{S}^{{\prime} }+\frac{\partial \,{p}_{{{\rm{CO}}}_{2}}}{\partial {A}_{{\rm{T}}}}{A}_{{\rm{T}}}^{{\prime} }+\frac{\partial \,{p}_{{{\rm{CO}}}_{2}}}{\partial {C}_{{\rm{T}}}}{C}_{{\rm{T}}}^{{\prime} },$$

where the four drivers are temperature *T*, salinity *S*, total alkalinity *A*_T_ and total dissolved inorganic carbon *C*_T_, the prime indicates a monthly anomaly relative to the annual mean, and the partial derivatives are the sensitivities of ${p}_{{{\rm{CO}}}_{2}}$ to each driver. Here this equation was used differently to distinguish the indirect effect of physical climate change (radiative effect) from the direct effect of increasing atmospheric CO_2_ on ocean chemistry (geochemical effect), assuming that the former affects the driver anomalies, whereas the latter affects the sensitivities. This climate–CO_2_ separation was used to distinguish the combined effects of the sensitivities from those of the driver anomalies in terms of how they affect the total change in ${p}_{{{\rm{CO}}}_{2}}^{{\prime} }$ between 2006–2015 and 2091–2100 (Methods). For brevity, analysis focused on the nine CMIP5 models. The results confirm that the change in sensitivities increases only the seasonal amplitude, whereas the change in driver anomalies alters the timing (Fig. 5). The effect from the change in the driver anomalies is roughly doubled after accounting for the synergy with the change in sensitivities (Extended Data Fig. 5). That synergy is strong enough in most models to produce a sign reversal in the basin-wide mean ${p}_{{{\rm{CO}}}_{2}}^{{\prime} }$ during at least part of summer. Thus it is the radiative effect that produces the change in timing.

This timing change also affects the seasonal amplitude and trends in seasonal means. With the timing change, the seasonal amplitude of ${p}_{{{\rm{CO}}}_{2}}^{{\prime} }$ doubles rather than triples during this century under RCP8.5 (Supplementary section 2). It also causes this century’s change in ${p}_{{{\rm{CO}}}_{2}}$ to be 20 ± 7% more in summer and 9 ± 2% less in winter, for seasonal averages, compared with no change in timing, that is, no physical climate change (Supplementary Section 3).

**Extended Data Fig. 6:**
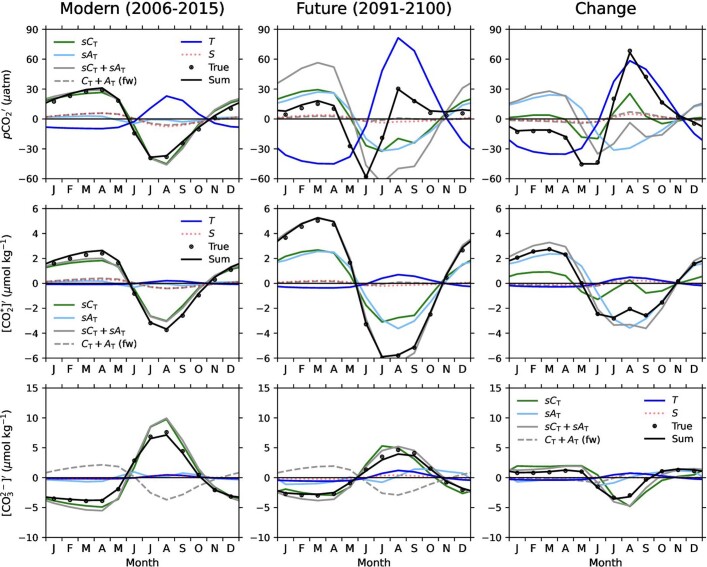
Thermally driven variations increase and become dominant for ${{\boldsymbol{p}}}_{{{\bf{CO}}}_{{\bf{2}}}}^{{\prime} }$, unlike for [CO_2_*]′ and [CO_3_^2−^]′. Contributions to seasonal variability deconvolved with the freshwater Taylor expansion are shown as climatologies of the CMIP5 mean under RCP8.5 for 2006– 2015 (left), 2091–2100 (middle), and their difference (right). Modern monthly variations in ${p}_{{{\rm{CO}}}_{2}}$ (top) are driven mostly by changes in *C*_T_, while in the future, thermally driven variations become dominant. Conversely, thermally driven variations are always small for [CO_2_*] (middle) and [CO_3_^2−^] (bottom), both now and in the future. Agreement between the simulated variable (black dots, True) and the sum of the components (solid black) indicates the consistency of the deconvolution.

To further distinguish the individual drivers, we used a freshwater Taylor expansion (equation (4), Methods) in the usual way (Supplementary Section 4). For the modern Arctic Ocean, the broad summer minimum is dominated by the *C*_T_ term, as in the subarctic^4,12,13,27^ (Extended Data Fig. 6). Conversely, this century’s reversal of summer ${p}_{{{\rm{CO}}}_{2}}^{{\prime} }$ found in most models is driven by the thermal term.

**Extended Data Fig. 7:**
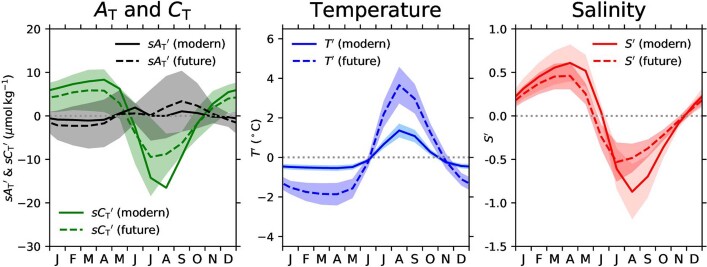
Reversal in summer ${{\boldsymbol{p}}}_{{{\bf{CO}}}_{{\bf{2}}}}^{{\prime} }$ is driven by a threefold increase in summer T′, while other changes are much smaller. Arctic domain averages of monthly anomalies of the CMIP5 mean under RCP8.5 are shown for the modern (2006–2015, solid) and future (2091–2100, dashed) climatologies for the four driving variables: *sA*_T_^′^ (cyan), *sC*_T_^′^ (green), *T*^′^ (blue), and *S*^′^ (red). The shading indicates model spread (±1 s.d., n = 9), which for *sA*_T_^′^ and *sC*_T_^′^ is shown only for the future.

The growing importance of the thermal term is not driven by the changes in the sensitivities of ocean ${p}_{{{\rm{CO}}}_{2}}$ to its driving variables. Although the sensitivity of ${p}_{{{\rm{CO}}}_{2}}$ to temperature nearly triples, ${p}_{{{\rm{CO}}}_{2}}$ sensitivities to *A*_T_ and *C*_T_ increase by about 30% more (Supplementary Fig. 11). Hence the main cause must be changes in driver anomalies. Indeed, summer *T*′ roughly triples, whereas the magnitude of salinity normalized *A*_T_′ (*sA*_T_′) changes relatively little and that of *C*_T_′ (*sC*_T_′) even declines (Extended Data Fig. 7). Although the effect of warming generally dominates future seasonal variations of ${p}_{{{\rm{CO}}}_{2}}$, it has little effect on those of other CO_2_ system variables except for [H^+^].

## Other CO_2_ system variables

**Extended Data Fig. 8:**
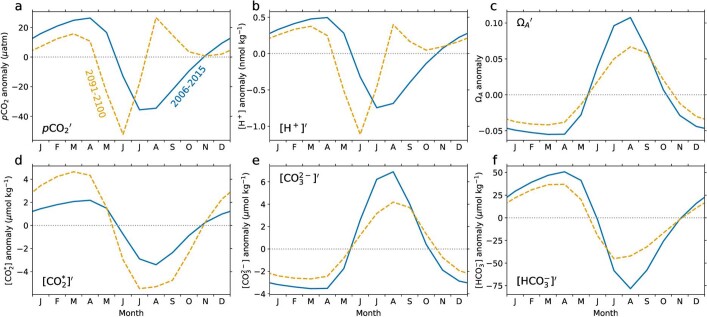
Changes in seasonal variations of ${{\boldsymbol{p}}}_{{{\bf{CO}}}_{{\bf{2}}}}$ and [H^+^] are similar but differ from other CO_2_ system variables. Climatologies of monthly anomalies for the CMIP5 mean under RCP8.5 averaged over the Arctic domain are shown for ${p}_{{{\rm{CO}}}_{2}}$ and other surface CO_2_ system variables for 2006–2015 (modern, blue solid) and 2091–2100 (future, orange dashed).

**Extended Data Fig. 9:**
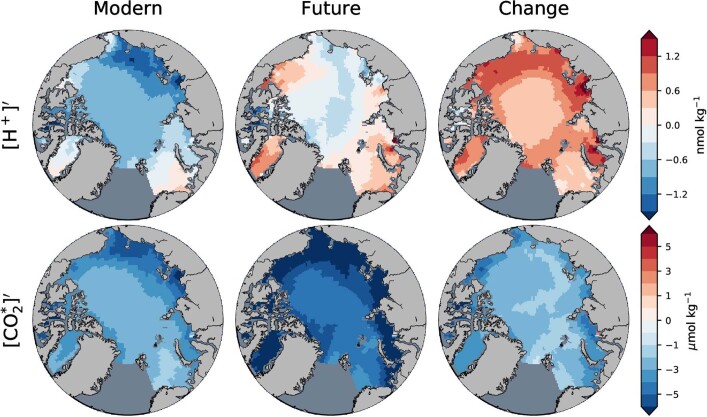
Summer [H^+^]^′^ reverses sign during this century mainly in the shelf seas, resembling ${{\boldsymbol{p}}}_{{{\bf{CO}}}_{{\bf{2}}}}^{{\prime} }$. Arctic maps of summertime anomalies of [H^+^] and [CO_2_*] for the CMIP5 mean under RCP8.5 are shown for climatologies of 2006–2015 (left, modern), 2091–2100 (middle, future), and their difference (right). The similarity of the sign reversal in summer anomalies of ${p}_{{{\rm{CO}}}_{2}}$ and [H^+^] is explained by their common sensitivity to temperature, while [CO_2_*] does not reverse sign because it remains relatively insensitive to temperature.

**Extended Data Fig. 10:**
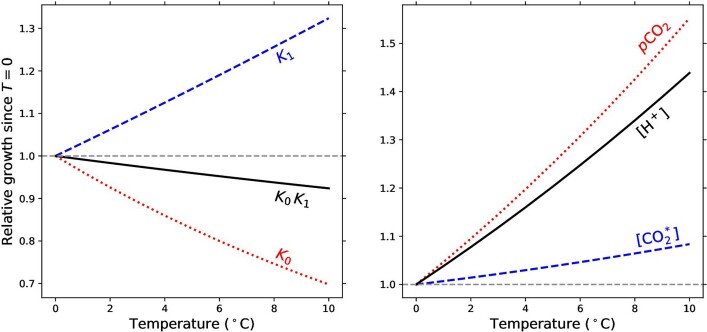
Seasonality of ${{\boldsymbol{p}}}_{{{\bf{CO}}}_{{\bf{2}}}}$ and [CO_2_*] differ because of the CO_2_ solubility *K*_0_, which depends on *T*. Shown is the relative effect of *T* on *K*_0_, *K*_1_, and their product as well as on [H^+^], ${p}_{{{\rm{CO}}}_{2}}$, and [CO_2_*]. Values between 0 to 10 °C were normalized by dividing by the value at 0 °C to compute their relative effect. All *K*_0_ and *K*_1_ are computed at *S* = 34. The CO_2_ system variables were computed with standard equilibrium calculations at the same salinity and temperatures as described in the Methods. Although not shown, [HCO_3_^−^] declines by 0.3% over the 0–10 °C range.

The annual cycle of [H^+^]′ and its projected changes in CMIP5 under RCP8.5 are much like those of ${p}_{{{\rm{CO}}}_{2}}^{{\prime} }$, suggesting a nearly linear relationship despite large changes in temperature (Extended Data Figs. 8 and 9). To clarify their ties, we combine Henry’s law ([CO_2_*] = *K*_0_ *C*_f_ ${p}_{{{\rm{CO}}}_{2}}$)^28^, with the first dissociation constant for carbonic acid (*K*_1_ = [H^+^][HCO_3_^−^]/[CO_2_*]) and rearrange to

2 $${p}_{{{\rm{CO}}}_{2}}=\frac{\left[{{\rm{H}}}^{+}\right]\left[{{\rm{HCO}}}_{3}^{-}\right]}{{C}_{{\rm{f}}}{K}_{0}{K}_{1}},$$

where [HCO_3_^−^] is the bicarbonate ion concentration, *K*_0_ is the CO_2_ solubility, *C*_f_ is the fugacity coefficient (which remains very near unity), and [CO_2_*] is the sum of aqueous [CO_2_] and [H_2_CO_3_]. At constant temperature, the relationship between ${p}_{{{\rm{CO}}}_{2}}$ and [H^+^] is nearly proportional because the denominator in equation (2) is a constant, and [HCO_3_^−^], the other term in the numerator, varies little relative to its large background concentration. But even as temperature changes, the relationship between ${p}_{{{\rm{CO}}}_{2}}$ and [H^+^] remains nearly linear because thermally driven changes in [HCO_3_^−^] are negligible, while those for *K*_0_ and *K*_1_ largely cancel (Extended Data Fig. 10). Thus similar to ${p}_{{{\rm{CO}}}_{2}}$, the projected change in seasonal timing of [H^+^] over this century enhances the summer basin-wide mean, by 15 ± 6% for that season’s mean (Supplementary Table 3) and 23 ± 8% for its extreme.

Conversely, changes in seasonal variations of [CO_2_*] do not resemble those for ocean ${p}_{{{\rm{CO}}}_{2}}$ (Extended Data Figs. 8 and 9) because of its weaker sensitivity to changes in temperature. For a closed system with no air–sea CO_2_ exchange, the relative sensitivity of ${p}_{{{\rm{CO}}}_{2}}$ to temperature is currently about seven times greater than that of [CO_2_*] (Extended Data Fig. 10), as determined by the solubility *K*_0_. As surface water warms, the CO_2_ solubility changes instantaneously as does ocean ${p}_{{{\rm{CO}}}_{2}}$.

Yet even in an open system, the effect of rapid warming between summer months cannot be fully compensated by air–sea CO_2_ equilibration, which has an average e-folding time of several months under current Arctic conditions. Thus ocean ${p}_{{{\rm{CO}}}_{2}}$ must change more quickly than does ocean [CO_2_*], which can further adjust only through exchange with the atmosphere. These contrasting tendencies are confirmed by Taylor-series expansions (Supplementary Section 4). Seasonal temperature variations largely affect ${p}_{{{\rm{CO}}}_{2}}^{{\prime} }$ and [H^+^]′, and thus pH′, but much less [CO_2_*]′ and [CO_3_^2−^]′, whose seasonal variations are controlled largely by variations in *C*_T_ and *A*_T_ (Extended Data Fig. 6).

## Biological impacts

Ocean acidification is expected to adversely affect life-sustaining processes across various groups of marine organisms^29,30^. In the Arctic Ocean, marine calcifying organisms may be the most sensitive, particularly pteropods, which already show signs of shell degradation^31^. However, some non-calcifiers also have vulnerable life stages, including those of two keystone species, the copepod *Calanus glacialis*^32^ and polar cod (*Boreogadus saida*)^33^, the main link between zooplankton and marine mammals, seabirds and other fish^34^. These organisms are sensitive to different CO_2_ system variables^35^.

In marine calcifiers, dissolution of CaCO_3_ depends on [CO_3_^2−^], whereas CaCO_3_ production may be only correlated with that variable. That is, many marine calcifiers appear to take up HCO_3_^−^, suggesting that increasing [HCO_3_^−^] from ocean acidification would stimulate calcification (Ca^2+^ + HCO_3_^−^ → CaCO_3_ + H^+^) provided the proton is removed from body fluids by acid–base regulation; conversely, increases in body fluid [H^+^] would inhibit calcification^36–38^. Thus the [HCO_3_^−^]/[H^+^] ratio in seawater and body fluids may be more physiologically relevant from a thermodynamic perspective; however, kinetic constraints still imply that [CO_3_^2−^] is a fundamental controlling variable^39^. Projected lower [CO_3_^2−^]′ and higher [H^+^]′ in summer are proportionally much larger than the increase in [HCO_3_^−^]′, relative to background levels (Extended Data Fig. 8), suggesting that the long-term decline in calcification in the Arctic will be sharper in summer months. In some calcifiers, high [CO_2_*] may also mediate the impacts of ocean acidification^40^.

For water-breathing animals, metabolic CO_2_ is produced in mitochondria and diffuses across membranes and epithelia until it is eventually released to seawater via the gills, but that efflux could slow under higher environmental CO_2_ levels until internal levels adjust^41^. Cross-gill transport is usually formulated in terms of the ${p}_{{{\rm{CO}}}_{2}}$ gradient based on Fick’s first law. Thus more positive summer ${p}_{{{\rm{CO}}}_{2}}^{{\prime} }$ during this century would result in further build-up of internal CO_2_ as needed to re-establish the gradient across the gills. Fish can partially compensate for such build-up by adjusting the chemistry of interior fluids, but that compensation is less efficient for larvae and young juveniles^41^. This analysis would benefit from also considering effects of warming on internal ${p}_{{{\rm{CO}}}_{2}}$ and the [CO_2_*] gradient, acid excretion across gills^41–43^ and respiratory plasticity^44^.

Ocean acidification is concerning, but it is warming that is likely to most affect Arctic marine organisms during this century. Between 1996–2005 and 2091–2100, the summer maximum surface temperature averaged over the Arctic shelf seas is projected to increase from 3.0 °C (after model debiasing) to 8.5 ± 1.2 °C in CMIP5 (RCP8.5, *n* = 9) and to 10.8 ± 2.7 °C in CMIP6 (SSP5-8.5, *n* = 18), beyond the upper thermal limits of certain fish and other fauna endemic to the Arctic^33,45,46^. Thermal vulnerability will be heightened further by simultaneous ocean acidification, particularly in summer, based on observations and experiments with boreal to Arctic crustaceans, bivalves and fish^33,46–50^.

Today, surface ${p}_{{{\rm{CO}}}_{2}}$ in the Arctic Ocean generally exhibits a broad summer minimum driven by the dominance of biological drawdown of carbon over warming, in both observations and models. Conversely, local observations from an Arctic polynya region exhibit a increase in ${p}_{{{\rm{CO}}}_{2}}$ of 110 µatm in early summer, prompted by the disappearance of seasonal ice cover and, among other factors, a 9 °C warming^19^. Thermally driven increases in summer ${p}_{{{\rm{CO}}}_{2}}$ are projected here to amplify and their dominance to become more widespread as atmospheric CO_2_ increases, seasonal sea ice recedes, and sea surface temperature rises much more in summer than winter, the opposite of surface atmospheric temperature^7^. Resulting changes in the seasonal amplitude and timing of ${p}_{{{\rm{CO}}}_{2}}$ will modulate the change expected from the trend in the mean state. The timing changes projected for ocean ${p}_{{{\rm{CO}}}_{2}}$ and [H^+^] cause their summer extremes to transition from annual lows to annual highs, enhancing their changes during this century by about one-fourth. Hence, in summer, when biological activity is greater and there is usually a seasonal reprieve, Arctic Ocean acidification may instead be elevated beyond the already large projected change in the mean state, thus also making organisms more vulnerable to heightened temperature.

## Methods

### Arctic domain

Following previous studies^7,51^, we adopt the definition of the Arctic Ocean domain as being bounded by four Arctic gateways: the Barents Sea Opening and the Fram, Davis and Bering straits. This domain excludes the Nordic seas, which remain largely ice free even in winter. Ocean grid points external to the domain are masked out, both when showing maps (masking in dark grey) and when computing integrated quantities such as averages over the Arctic domain (basin-wide mean) or the Arctic’s individual regional seas.

### Earth system models

To assess potential changes while accounting for regional differences, physical climate change, and the carbon cycle, we used a suite of 9 ESMs that participated in CMIP5 and 18 ESMs that participated in CMIP6 (Supplementary Table 1). Using models from both phases improves statistical robustness and takes advantage of improvements in the community of models over the past decade^52^ while providing a test to check whether conclusions hold across model generations. One improvement in CMIP6 that could be important for the Arctic is that some models have much finer lateral resolution.

The CMIP models were used to assess monthly variations and trends in surface ocean ${p}_{{{\rm{CO}}}_{2}}$ and related surface-water ocean CO_2_ system variables. Monthly means of CMIP5 CO_2_ system variables were previously computed^3^ from monthly mean model output for total dissolved inorganic carbon *C*_T_, total alkalinity *A*_T_, temperature *T*, salinity *S*, total dissolved inorganic phosphorus *P*_T_ and total dissolved silicon Si_T_ using mocsy^53^. For CMIP6, the only CO_2_ system variable analysed was surface ocean ${p}_{{{\rm{CO}}}_{2}}$, which was provided by each model group. The CMIP5 results for 1860–2005 (historical experiment) were combined with three projections for 2006–2100 under the RCPs that reach radiative forcings of 2.6 W m^−2^, 4.5 W m^−2^ and 8.5 W m^−2^ (rcp26, rcp45 and rcp85 experiments), referred to as RCP2.6, RCP4.5 and RCP8.5, respectively. Likewise, the CMIP6 results for 1850–2014 (historical experiment) were combined with those for four projections for 2015–2100 under the Shared Socioeconomic Pathways (SSPs) that reach radiative forcings of 2.6 W m^−2^, 4.5 W m^−2^, 7.0 W m^−2^ and 8.5 W m^−2^ (ssp126, ssp245, ssp370 and ssp585 experiments), referred to as SSP1-2.6, SSP2-4.5, SSP3-7.0 and SSP5-8.5, respectively.

Fields were regridded to a 1° × 1° regular grid for model evaluation and comparison. Monthly mean anomalies relative to the annual mean were computed by subtracting a cubic-spline fit^54^ of the monthly mean times series at each grid cell. Decadal mean climatologies were compared between either 1996–2005 and 2091–2100 or 2006–2015 and 2091–2100, and tendencies were assessed as a function of the atmospheric CO_2_ level. The driving mechanisms were assessed with (1) Taylor-series expansions to quantify contributions from each of the four main input variables (*C*_T_, *A*_T_, *T* and *S*) and (2) idealized scenarios from CMIP5 with multiple simulations under different forcing to separate the direct chemical consequences attributable to the increase in atmospheric CO_2_ (geochemical effect) from the indirect consequences of physical climate change (radiative effect). Error bars given in the text are reported as ±1 s.d about the multimodel mean.

### Model evaluation

CMIP5 and CMIP6 seasonal climatologies constructed from the 1996–2005 model output years of each historical experiment were compared over the Arctic Ocean domain to observation-based products of surface ocean ${p}_{{{\rm{CO}}}_{2}}$ (refs. ^6,55^), sea surface temperature^56^ and sea-ice concentration^57^. Both the ${p}_{{{\rm{CO}}}_{2}}$ and sea-ice concentration^57^ data products are provided on a 0.25° × 0.25° regular latitude–longitude grid. For comparison, these data products and all model fields were regridded to the same World Ocean Atlas 1° × 1° regular latitude–longitude grid, that is, that of the sea-surface-temperature data product. Regridding was done using the nearest-neighbour algorithm in the cdo package (cdo remapnn)^58^. A common land mask was applied from the World Ocean Atlas over the Arctic Ocean domain.

### Debiasing

Models were debiased only to compute the maximum summer temperature in the shelf seas for 2091–2100 under RCP8.5 as well as under SSP5-8.5. The individual models were debiased by computing the 1996–2005 climatology for each model and then subtracting from that the observational climatology^56^ to obtain the model bias. The bias, at each grid point and month, was then removed from the 2091–2100 climatology. Then the annual maxima were computed and the area-weighted average taken over all grid points in the Arctic shelf seas (bottom depths <500 m). Results are reported as the CMIP5 and CMIP6 multimodel means ±1 s.d. In the same way, the observational database was masked and the maxima computed to obtain the modern data-based reference for the area-weighted summer maximum for the Arctic shelf seas. For other analyses, models were not debiased.

### Idealized experiments

Three of the CMIP5 models each provided a set of three idealized experiments: 1pctCO2, esmFixClim1 and esmFdbk1. All three experiments are forced by atmospheric CO_2_ that increases at the same rate, 1% per year (doubling after 70 years and quadrupling after 140 years, both relative to the pre-industrial level), but how that is felt by the Earth system differs. The 1pctCO2 simulation considers both the direct ‘geochemical’ effect of increasing CO_2_ on the carbon cycle and the ‘radiative’ effect of CO_2_ on physical climate, which drives physical changes, thereby affecting the carbon cycle indirectly. The esmFixClim1 simulation has identical forcing but considers only the direct effect (geochemical), whereas the esmFdbk1 simulation considers only the indirect effect (radiative).

These CMIP5 idealized experiments allow one to deconvolve the geochemical and radiative contributions, but they also come with limitations. The most obvious is that the rate of increase in atmospheric CO_2_ is larger than in the historical and high-end RCP8.5 scenarios. Second, the separation between the three experiments is imperfect. In the esmFixClim1 simulation, intended to eliminate physical climate change effects, there are slight increases in ocean temperature linked to the response of the terrestrial biosphere, because the higher CO_2_ reduces stomatal conductance, thus causing greater surface fluxes of sensible heat. Third, only three models provided results for the full set of experiments, and only two of those (IPSL-CM5A-LR and MPI-ESM-LR) continued the 1% per year atmospheric CO_2_ increase until the end of the 140-year simulation, whereupon atmospheric CO_2_ had quadrupled relative to the pre-industrial starting point (284 ppm). Conversely, the third model (GFDL-ESM2M) stopped that increase after the first 70 years, the point at which atmospheric CO_2_ had doubled, holding the same level over the remaining 70 years. Given the limited number of models, no attempt was made to provide quantitative estimates of model uncertainty.

### Climate–CO_2_ Taylor separation

As ${p}_{{{\rm{CO}}}_{2}}^{{\prime} }=f(T,S,{A}_{{\rm{T}}},{C}_{{\rm{T}}})$, a Taylor-series expansion yields equation (1), after neglecting second-order terms, covariances and minor contributions from *P*_T_ and Si_T_. Typically, that equation is used to compare contributions from each of the four drivers^12^, but here we use it in a broader way to distinguish the effects of the atmospheric CO_2_ increase from those of physical climate change. Thus the effects from the combined sensitivities were separated from those of the combined driver anomalies to ascribe general causes for differences between the modern reference state, defined as the 2006–2015 decadal climatology of surface ${p}_{{{\rm{CO}}}_{2}}^{{\prime} }$, and the future state, defined as the 2091–2100 climatology. Mathematically, that separation takes the following form

3 $$\Delta {p}_{{{\rm{CO}}}_{2}}^{{\prime} }=\left({\sum }_{i=1}^{4}\Delta {{\boldsymbol{\gamma }}}_{i}\begin{array}{c}{\times {\bf{X}}}_{i,0}^{{\prime} }\end{array}\right)+\left({\sum }_{i=1}^{4}{{\boldsymbol{\gamma }}}_{i,0}\begin{array}{c}{\times \Delta {\bf{X}}}_{i}^{{\prime} }\end{array}\right)+\left({\sum }_{i=1}^{4}\Delta {{\boldsymbol{\gamma }}}_{i}\begin{array}{c}{\times \Delta {\bf{X}}}_{i}^{{\prime} }\end{array}\right),$$

where the prime is the monthly anomaly, Δ is the change between the modern (2006–2015) and future (2091–2100) decades, the 0 subscript refers to the modern decade, and two vectors represent the four drivers **X** = (*T,* *S,* *A*_T_*,* *C*_T_) and the corresponding sensitivities **γ** = (∂${p}_{{{\rm{CO}}}_{2}}$/∂*T*, ∂${p}_{{{\rm{CO}}}_{2}}$/∂*S*, ∂${p}_{{{\rm{CO}}}_{2}}$/∂*A*_T_, ∂${p}_{{{\rm{CO}}}_{2}}$/∂*C*_T_). The first right-hand side term (in parentheses) in equation (3) characterizes the effect of increasing atmospheric CO_2_ (without physical climate change), which affects the sensitivities, whereas the second and third terms (each in separate parentheses) would be null without the effect of physical climate change, which affects the driver anomalies.

In practice, the normal Taylor expansion for the modern state is made using modern sensitivities and driver anomalies, whereas that for the future state is made using future sensitivities and driver anomalies. The total difference between those two states is due to changes in both the sensitivities and the driver anomalies. To isolate the effect of the changes in the sensitivities (without physical climate change), the sum of the four terms in equation (1) is computed using the future sensitivities with the modern driver anomalies and then the modern reference state is subtracted to get the perturbation (ΔSensitivities, first term in equation (3)). Likewise, the effect of the changes in driver anomalies is computed using the modern sensitivities with the future driver anomalies and then subtracting the modern state (ΔAnomalies, second term in equation (3)). However, the sum of the modern reference state and those two perturbations does not add up to the future state because it does not account for the synergy between the change in driver anomalies and the increase in sensitivities (third term in equation (3)). Thus that synergy is accounted for along with the change in driver anomalies (ΔAnomalies*, second and third terms in equation (3)) by subtracting the state computed with only increased sensitivities (first term) from the future state (all three terms).

This climate–CO_2_ separation is a simplification of a more elaborate regrouping of terms that was derived to analyse contributions to the amplitude of the annual cycle of ${p}_{{{\rm{CO}}}_{2}}$ in the CMIP5 models in a study^4^ that excluded the Arctic Ocean and did not address seasonal timing. Nor have previous studies emphasized that changes in sensitivities come mainly from the increase in atmospheric CO_2_, whereas changes in driver anomalies come from physical climate change. This climate–CO_2_ Taylor-series expansion requires results from just one model experiment. Hence, we were able to use it here to assess all nine CMIP5 models forced under the RCP8.5 scenario, unlike the approach described in the previous section that relies on multiple idealized simulations carried out with less realistic forcing and for which only two CMIP5 models have provided a complete set of results.

### Freshwater Taylor-series expansion

To assess the contributions to ${p}_{{{\rm{CO}}}_{2}}$ variations from individual terms, a Taylor expansion that accounts for effects from freshwater fluxes is adopted. After normalizing the *A*_T_ and *C*_T_ terms in equation (1) to a reference salinity *S*_0_, ref. ^59^ noted that interannual variations in ${p}_{{{\rm{CO}}}_{2}}$ driven by the normalized *A*_T_ term became negligible whereas the normalized *C*_T_ term declined, essentially becoming equal to the sum of the last two terms in equation (1), which are not normalized. Building on that finding and the work of ref. ^60^, ref. ^61^ introduced a modified equation that separated out the effects on *A*_T_ and *C*_T_ into those that are biogeochemically driven and those that are physically driven from variations in freshwater fluxes (precipitation minus evaporation, river input, and sea-ice melt and formation). Thus equation (1) can be rewritten as

4 $${p}_{{{\rm{CO}}}_{2}}^{{\prime} }\approx \frac{\partial {p}_{{{\rm{CO}}}_{2}}}{\partial T\,}{T}^{{\prime} }+\frac{\partial {p}_{{{\rm{CO}}}_{2}}}{\partial S\,}{S}^{{\prime} }+\left[\frac{{A}_{{\rm{T}}}}{S}\frac{\partial {p}_{{{\rm{CO}}}_{2}}}{\partial {A}_{{\rm{T}}}}{S}^{{\prime} }+\frac{{C}_{{\rm{T}}}}{S}\frac{\partial {p}_{{{\rm{CO}}}_{2}}}{\partial {C}_{{\rm{T}}}}{S}^{{\prime} }\,\right]+\left(\frac{S}{{S}_{0}}\frac{\partial {p}_{{{\rm{CO}}}_{2}}}{\partial {A}_{{\rm{T}}}}\,{\rm{s}}{A}_{{\rm{T}}}^{{\prime} }+\frac{S}{{S}_{0}}\frac{\partial {p}_{{{\rm{CO}}}_{2}}}{\partial {C}_{{\rm{T}}}}\,s{C}_{{\rm{T}}}^{{\prime} }\right),$$

where *sA*_T_ and *sC*_T_ are the salinity-normalized quantities (*sX* = *XS*_0_/*S*). In our case, *S*_0_ is the annual mean salinity in each grid cell because the focus is on monthly anomalies relative to the annual mean, a choice also adopted previously^1^ that should minimize known problems with salinity normalization^62^. Thus each of the two original terms for *A*_T_ and *C*_T_ in equation (1) are split into two components: one driven by variations in salinity (freshwater fluxes, in square braces) and another driven by variations in salinity-normalized quantities (biogeochemical, in parentheses).

Many subsequent studies have used this freshwater Taylor expansion. However, the *S*/*S*_0_ ratio before the two terms in parentheses was subsequently dropped^63^, a simplification that is often adopted^1,64–66^. That is, the *S*/*S*_0_ ratio is assumed to be equal to 1, for example, for seasonal anomalies relative to the annual mean^1^. Here this simplification is avoided because in the Arctic Ocean, substantial short-term variations in salinity are expected.

In practice, the deconvolution was performed locally and resulting terms were area-weighted for basin-wide averages. The partial derivatives (sensitivities) were computed numerically using derivnum^67^ from mocsy^53^. For the 2006–2015 mean, we adopted a basic approach: for each term and month, the monthly mean anomaly relative to the annual mean was computed and multiplied by the average of the corresponding monthly mean and annual-mean sensitivities. The sum of all terms generally agreed well with the actual simulated variable (for example, ${p}_{{{\rm{CO}}}_{2}}$) for that modern decadal average. Conversely, at the end of the century under RCP8.5 (2091–2100), the basic approach led to poor agreement when there were dramatic changes between months, such as between the ${p}_{{{\rm{CO}}}_{2}}$ minimum in early summer and its maximum in late summer. To improve agreement, we revised the approach for the end-of-century deconvolution following three steps: (1) anomalies were instead computed for each month between consecutive years (between Januaries, between Februaries and so on) and multiplied by the corresponding average sensitivity between each pair of years; (2) each of those products (for each month and each term) were then summed up across years to have a decomposition of the total change between the modern and future decades; and (3) finally, the total change for each term and month were added to each term of the monthly deconvolution for 2006–2015 to obtain the deconvolution for 2091–2100. Agreement for that decade then became similar to that found when using the basic approach for 2006–2015.

### Spatial average timing of highs and lows

Plots are shown detailing the evolution in timing of the annual high and low for multiple variables. That timing (month of annual high and low) is represented as an average across a basin or region, which is computed in one of two ways. For monthly anomalies of ${p}_{{{\rm{CO}}}_{2}}$ and other variables, the area-weighted mean of the variable was first computed for each month of the annual-cycle decadal climatology, and then the months of the maximum and minimum were selected from the resulting 12 points. This approach gives less weight to regions with low monthly anomalies such as in ice-covered regions for ${p}_{{{\rm{CO}}}_{2}}^{{\prime} }$. A second approach was used for sea-ice retreat and growth dates, defined as when sea-ice concentration first drops below 0.15 and when it first rises back above 0.15, respectively. In this case, the timing (month index) at the different grid cells was recorded and used to compute the area-weighted mean month index. When showing the evolution of this timing of annual highs and lows as a function of increasing atmospheric CO_2_, curves were fit with a cubic spline to suppress interannual variations.

### CO_2_ system equilibrium calculations

Equilibrium calculations for [H^+^], ${p}_{{{\rm{CO}}}_{2}}$ and [CO_2_*] shown in Extended Data Fig. 10 were made with mocsy^53^ and the constants recommended for best practices with *A*_T_ = 2,130 µmol kg^−1^ and *C*_T_ = 2,000 µmol kg^−1^. Total dissolved inorganic phosphorus and silicon were set to zero.

## Online content

Any methods, additional references, Nature Research reporting summaries, source data, extended data, supplementary information, acknowledgements, peer review information; details of author contributions and competing interests; and statements of data and code availability are available at 10.1038/s41586-022-05205-y.

## Supplementary information

Supplementary Information Supplementary discussion, figures, tables and references.

Peer Review File

## Data Availability

Output from the CMIP5 and CMIP6 Earth system models used in this study is available for download from the Earth System Grid Federation, for example, from the node at Lawrence Livermore National Laboratory (https://esgf-node.llnl.gov/projects/esgf-llnl/). The gridded observation product of surface ocean temperature is from the World Ocean Atlas 2018^56^ and is available at https://www.ncei.noaa.gov/products/world-ocean-atlas. The gridded observation product for sea-ice concentration from the National Snow and Ice Data Center (NSIDC), referred to as Gridded Monthly Sea Ice Extent and Concentration, 1850 Onward, Version 2 from ref. ^57^, is available at https://nsidc.org/data/g10010. The gridded observation product for surface ocean ${p}_{{{\rm{CO}}}_{2}}$ from refs. ^6,55^ is available at https://accession.nodc.noaa.gov/0209633 and that from ref. ^2^, which was used only in Supplementary Fig. 1b, is taken from their supplementary information.

## Code availability

For analysis, we used open-source Python, versions 2.7 and 3, namely the numpy, scipy, pandas and matplotlib modules. To regrid model data, we used climate data operators available at https://code.mpimet.mpg.de/projects/cdo/. To compute ocean CO_2_ system variables and their sensitivities to driving variables, we used mocsy^53,67^, available from https://github.com/jamesorr/mocsy. To detrend and smooth data, we relied on a fast cubic spline^54^ translated for use in Python and available at https://github.com/jamesorr/fastspline.
