# Supplementary material for: Arctic Ocean annual high in pCO2 could shift from winter to summer
Source: Nature. 2022 Oct 5;610(7930):94–100. doi: 10.1038/s41586-022-05205-y (PMC9534769; doi:10.1038/s41586-022-05205-y)
Supplement: Supplementary file 1 — Supplementary discussion, figures, tables and references. [file 41586_2022_5205_MOESM1_ESM.pdf]

nature portfolio

https://doi.org/10.1038/s41586-022-05205-y

Supplementary information

Arctic Ocean annual high in p_{CO_2} could shift from winter to summer

In the format provided by the authors and unedited

Supplementary Information

1 Idealized separation

Three idealized experiments were provided by each of three CMIP5 models. The three experiments were all forced under an atmospheric CO_2 increase of 1% per year, but they separately accounted for the geochemical effect, the radiative effect, or their combination (see Methods). The geochemical effect is computed from the *esmFixClim1* experiment where there is no effect of increasing atmospheric CO_2 on the radiative balance (no physical climate change), only its effect on the carbon cycle. That effect amplifies the simulated seasonal cycle of $p\mathrm{CO}_2'$ without changing its timing; it never produces a sign change. Conversely, the radiative effect is computed from the *esmFdbk1* experiment, where there is no effect of increasing atmospheric CO_2 on the carbon cycle, only its effect on the radiative balance. Thus it simulates physical climate change but not how ocean chemistry is affected by the atmospheric CO_2 increase.

In the full experiment (*1pctCO2*), which includes the effect of increasing atmospheric CO_2 on both the carbon cycle and climate, the summer $p\mathrm{CO}_2'$ becomes less negative with time in all models (Supplementary Fig. 12). That change is in part due to the change from the radiative effect, but that alone does not reverse the sign of the anomaly since the change from the opposing geochemical effect is stronger. The remaining factor is that the radiative effect is amplified due to the higher sensitivity of $p\mathrm{CO}_2$ to changes in physical climate change variables (T and S) at higher atmospheric CO_2, a synergy that is absent from the *esmFixClim1* and *esmFdbk1* experiments. The change from that amplified radiative effect, computed as the difference between the *1pctCO2* and *esmFixClim1* experiments, outweighs that from the geochemical effect and reverses the sign of $p\mathrm{CO}_2'$ in the 2 models where the atmospheric CO_2 forcing reaches a quadrupling; in the remaining model (GFDL-ESM2M), atmospheric CO_2 reaches only a doubling. Despite the limited set of results and the idealized forcing, this assessment reveals that the sign reversal of $p\mathrm{CO}_2'$ is consistently steered by physical climate change and amplified by higher levels of atmospheric CO_2.

2 Effect on seasonal amplitude

The change in timing also affects the seasonal amplitude. Considering only changes in sensitivities (no timing change) between the 2006–2015 and 2091–2100 decades under RCP8.5, the Arctic Ocean's basin-wide mean of the seasonal amplitude of $p\mathrm{CO}_2'$ would have increased by $204 \pm 86\%$, a tripling relative to modern conditions. Yet the projected seasonal amplitude actually increases by $100 \pm 87\%$, being damped by changes in the monthly anomalies. Although still substantial, that doubling is ten times less than reported by a previous study[2], which emphasized local changes and ignored physical climate change; conversely, our reanalysis of those observation-based results (Supplementary Fig. 1b) indicates that the basin-wide average of the amplitude under RCP8.5 roughly triples, in line with the geochemical effect assessed above that considers only changes in sensitivities.

3 Effect on seasonal trend

The projected difference in Arctic Ocean pCO_2 between 2006–2015 and 2091–2100 is 519 $\pm$ 10 μatm in CMIP5 under RCP8.5 for the climatological annual mean but differs for the summer and winter means. The difference in summer means is 9$\pm$4% higher, but if one would consider only the increase in the pCO_2 sensitivities (geochemical effect), it would be 11 $\pm$ 9% lower (Fig. 5). Thus the radiative effect under RCP8.5 increases the change in the basin-wide mean of average summer pCO_2 by 20 $\pm$ 7% (100 $\pm$ 40 μatm) by altering driver anomalies and hence the seasonal timing (Supplementary Table 3). Larger still is the corresponding enhancement in summer extremes, i.e., 29 $\pm$ 9% (150 $\pm$ 50 μatm) between the summer low in 2006–2015 and the summer high in 2091–2100. In most models, the increase in summer pCO_2 from the radiative effect is enough to overwhelm the decrease from heightened sensitivities (geochemical effect) even when combined with the already negative summer anomaly of 2006–2015, making the 2091–2100 anomaly positive during part of summer (Extended Data Fig. 5).

For the change in winter, the tendencies are opposite but small enough that the winter mean anomaly generally remains positive. The difference in winter mean pCO_2 anomalies between 2006–2015 and 2091–2100 is 3 $\pm$ 4% lower than the annual-mean difference, but it would be 6 $\pm$ 4% higher with no change in driver anomalies, only an increase in sensitivities. Thus physical climate change reduces the change in the winter mean pCO_2 by 9 $\pm$ 2% relative that of the annual-mean. In both winter and summer then, the radiative effect dominates the geochemical effect, but this does not indicate which driving variables are responsible.

4 Contributions of individual drivers

To assess contributions of individual drivers, a Taylor expansion was used in the conventional way after separating out freshwater-driven variations in A_T and C_T (equation (4), Methods). Those freshwater terms (in square braces) are generally the largest terms, but their effects on pCO_2' usually cancel one another (Extended Data Fig. 6). After isolating their contributions, the biogeochemically driven variations in salinity-normalized A_T and C_T become apparent. Although smaller, they tend not to cancel. For the modern Arctic Ocean, the salinity-normalized C_T term dominates over the corresponding A_T term and the opposing T term, producing a broad summer minimum in pCO_2'. Likewise, the C_T term is known to dominate in the subarctic[4,12,13,27].

More novel is how contributions change in the future projection. Unlike for the 2006–2015 balance, during 2091–2100 in CMIP5 under RCP8.5 the relative importance of the salinity-normalized A_T term generally grows and must be accounted for along with the salinity-normalized C_T term (Extended Data Fig. 6). The cause appears to be in the changing balance of the corresponding anomalies, with the magnitude of sC_T' declining more than that of sA_T' (Extended Data Fig. 7). Just before summer, the effect of $C_T + A_T$ still tends to dominate in the models, typically producing a minimum in June. Yet in all models that minimum is sharpened by the stronger increase in the thermal term, which begins in late spring and peaks in August (Supplementary Fig. 13). That steep increase also drives the corresponding sign reversal of basin-wide mean summer pCO_2' during this

century seen in most models. The two models that fail to reverse sign during summer (JAS) have the weakest thermal terms and the strongest combined $A_T + C_T$ terms, but even they reach positive $pCO_2{}'$ by October. The future dominance of the thermal component of $pCO_2{}'$ is particularly prominent in the shelf seas (Fig. 4).

As for other CO_2 system variables, they are not all affected in the same way by the various drivers (Extended Data Fig. 6). Seasonal temperature variations largely affect $pCO_2{}'$ and $[H^+]'$ (and thus pH′), but much less $[CO_2^*]'$, whose modern and future variations are controlled largely by variations in salinity-normalized A_T and C_T. Contributions to those four variables from variations in salinity normalized A_T are currently negligible but by 2100 grow to rival those from salinity normalized C_T. Their contributions from the freshwater component (salinity-driven changes in $A_T + C_T$) are always negligible, unlike for $[CO_3^{2-}]'$ and the saturation state Ω', which are only weakly sensitive to seasonal temperature changes, being closely tied to CO_2^* through two basic equations: $CO_2^* + CO_3^- + H_2O \rightleftharpoons 2HCO_3^-$ and $\Omega = [Ca^{2+}][CO_3^{2-}]/K_{sp}$.

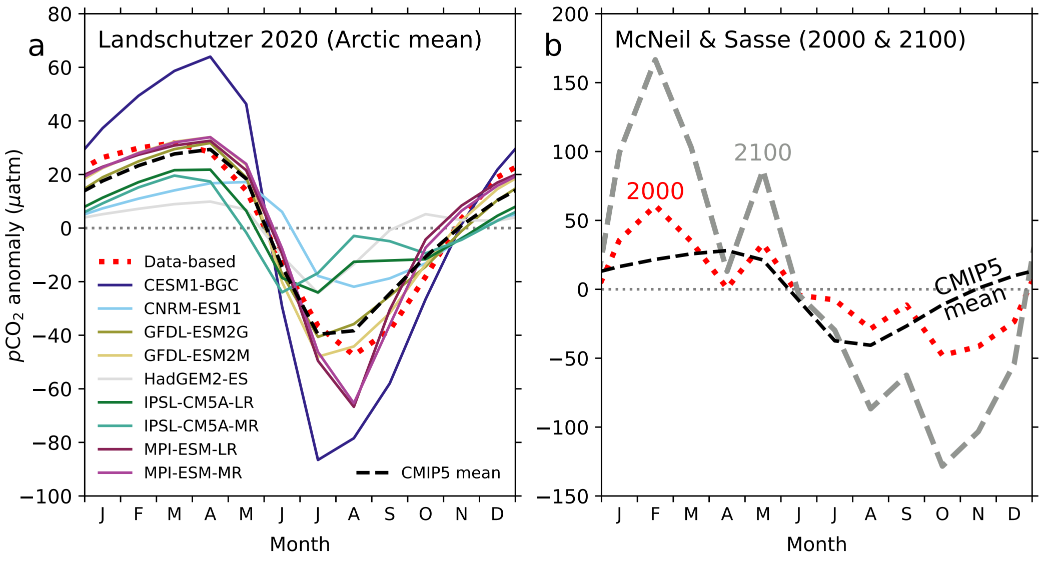

Supplementary Fig. 1 | **Models and observations agree that Arctic Ocean $p\mathrm{CO}_2'$ is lower in summer than in winter.** The basin-wide average of $p\mathrm{CO}_2'$ over the Arctic Ocean in the CMIP5 models (2006–2015 climatology under RCP8.5) is compared to two neural network-based, observational products (red dots): (a) a high-resolution monthly climatology for the open and coastal ocean[6,55] based on surface $p\mathrm{CO}_2$ measurements collected over 1998–2015[68] and (b) an earlier estimate[2] based on $p\mathrm{CO}_2$ calculated from neural-net-derived, monthly climatologies of C_T and A_T, each from $p\mathrm{CO}_2$ calculated for year 2000. The latter also includes an extrapolation for year 2100, where C_T is assumed to be affected by the increase in atmospheric CO_2 (RCP8.5 scenario) while physical climate change is ignored. Lines represent area-weighted results for all grid cells over the Arctic domain. Before averaging, the observation-based products and the detrended 2006–2015 modelled climatologies (RCP8.5) were interpolated a 1°x1° grid. A common mask was applied between each data product and each of the CMIP5 models (coloured solid lines) and their mean (dashed black line).

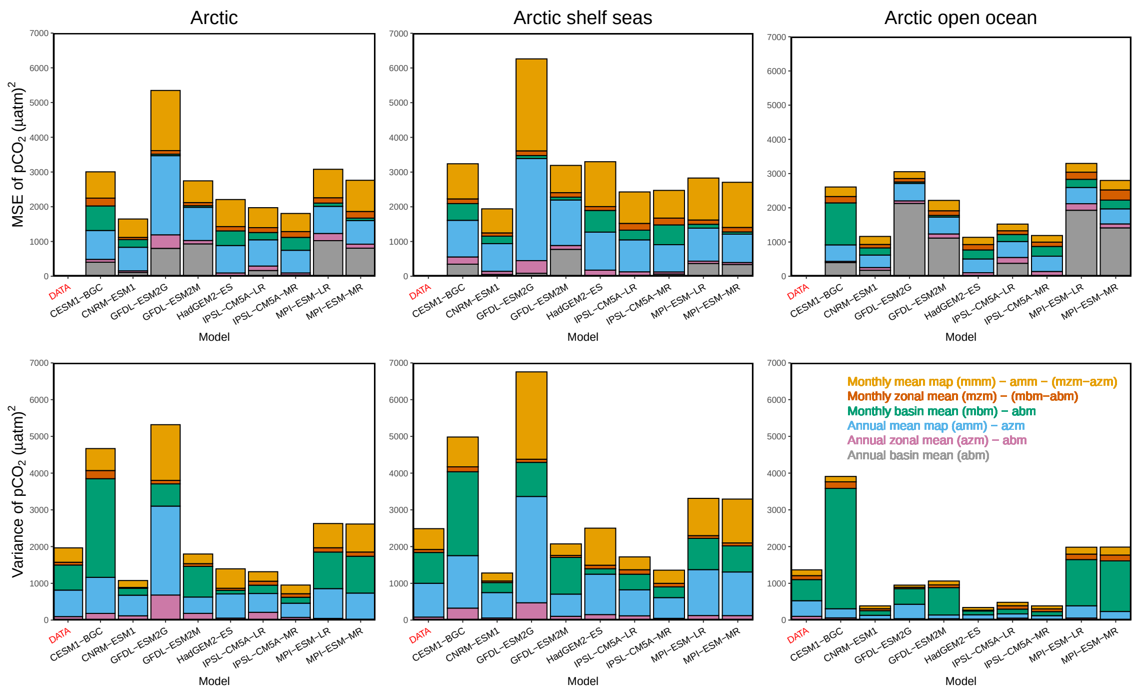

Supplementary Fig. 2 | **Much of the simulated overall seasonal variability is characterized by the basin varying as a whole, comparing well with observations.** The CMIP5 models are compared over the Arctic domain to a recent global observation-based product[55]. Following Gleckler et al.[69], the mean square error (MSE) is separated into orthogonal spatiotemporal components: (1) the annual mean basin mean, (2) the annual mean zonal mean (minus the annual mean basin mean), (3) the annual mean map (minus the annual zonal mean), (4) the annual cycle of the basin mean (minus the annual mean basin mean), (5) the annual cycle of the zonal mean (minus the annual zonal mean and the previous component), and (6) the annual cycle of the surface map (minus the annual mean map and monthly deviations of the zonal mean relative to its annual mean). Here we also distinguish the contribution from the annual cycle of the basin mean (4), whereas previously that has been combined with (5). The same orthogonal separation is made for the variance of $p\mathrm{CO_2}$, for which the first component has no variance by definition.

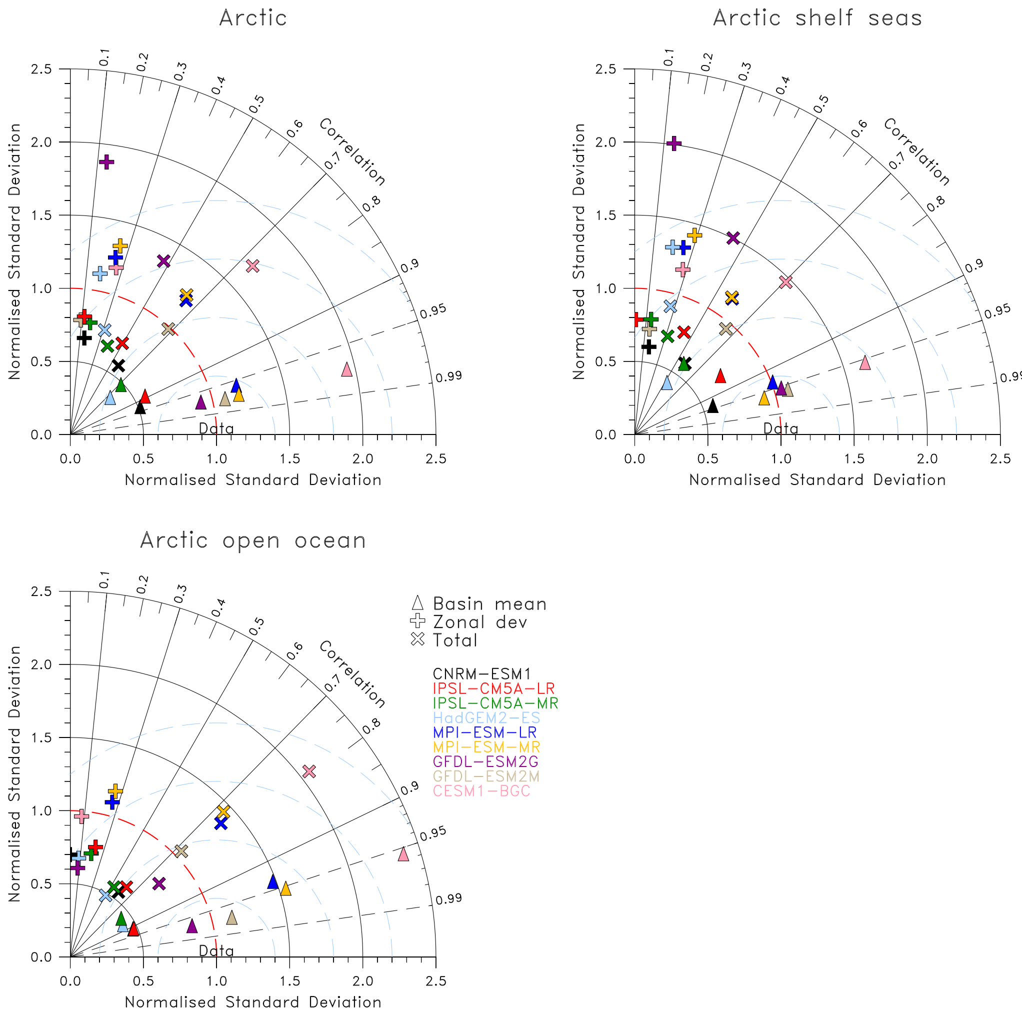

Supplementary Fig. 3 | **The basin-wide mean component of overall seasonal variability of *p*CO_2 correlates well with observations.** Taylor diagrams for CMIP5 seasonal variations in Arctic Ocean *p*CO_2 quantify agreement with a observation-based reference[6]. Colours indicate individual models, while shapes represent space-time components. The total seasonal component (x) is computed from maps of monthly anomalies (monthly mean minus annual mean) and is further separated into two orthogonal components: (1) triangles: monthly anomalies of the basin-wide mean (monthly basin-wide means minus annual-mean basin mean) and (2) pluses: residual monthly anomalies (maps of monthly anomalies minus the monthly basin-wide mean anomalies).

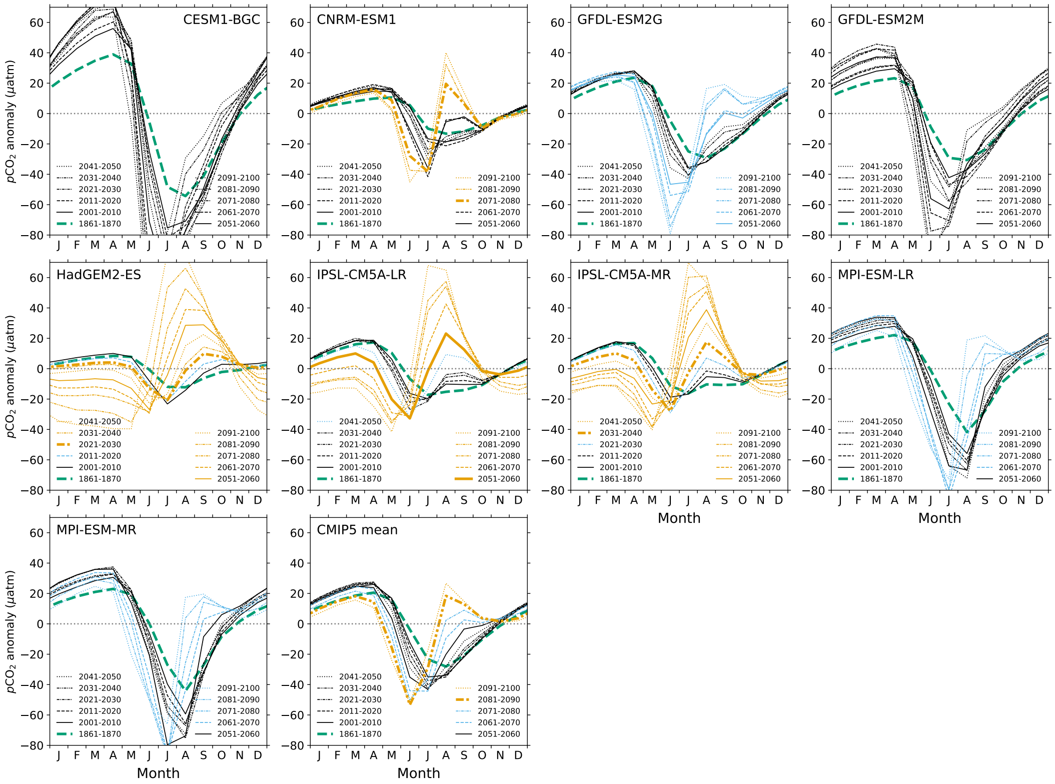

Supplementary Fig. 4 | **Seasonal timing of the CMIP5 models evolves gradually ending at different points along a common pathway.** Plots are as in Fig. 3c for the CMIP5 mean except here are shown for the individual CMIP5 models.

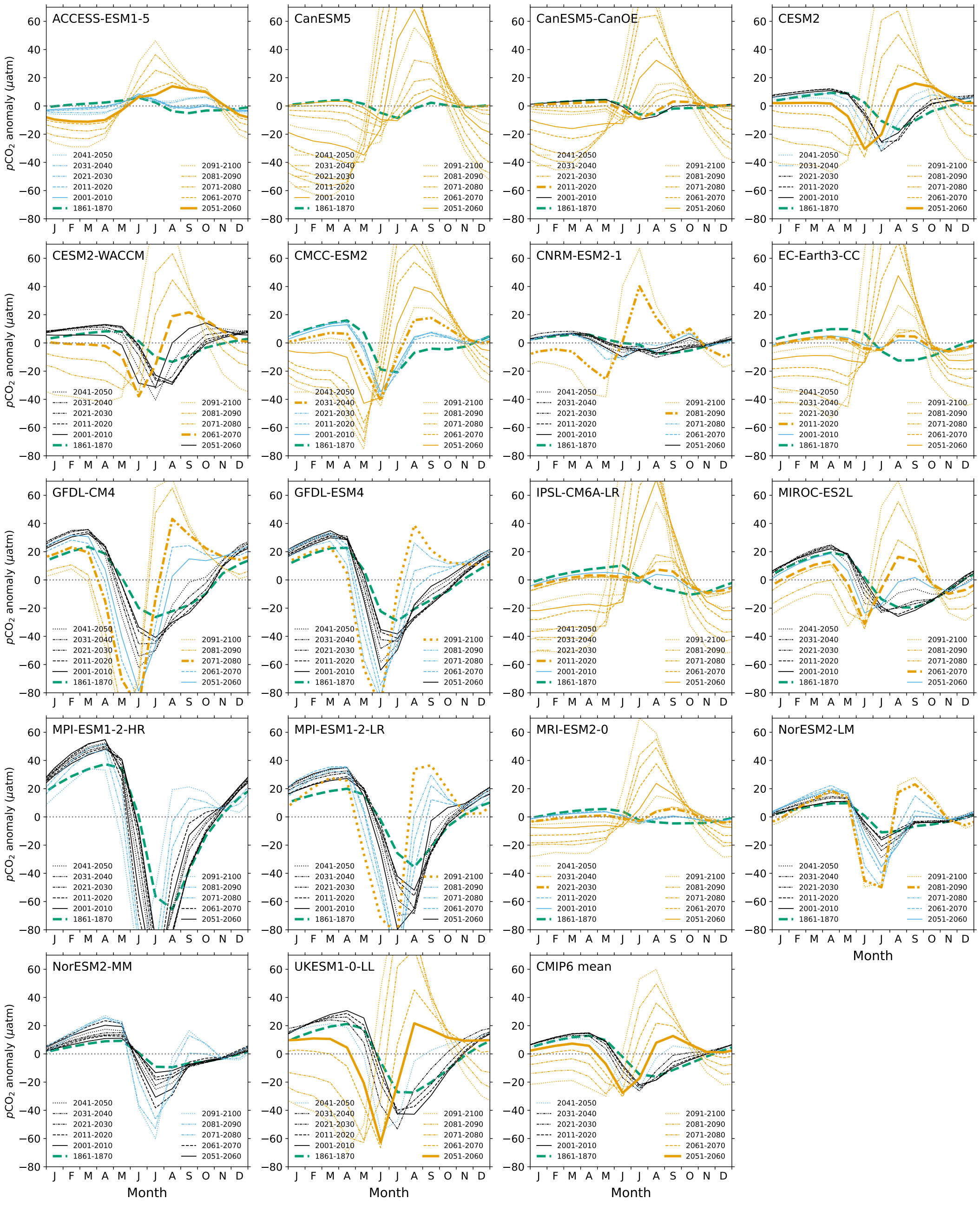

Supplementary Fig. 5 | **Seasonal timing of the CMIP6 models evolves gradually ending at different points along a common pathway.** Plots are as in Fig. 3d for the CMIP6 mean except here also shown for the individual CMIP6 models.

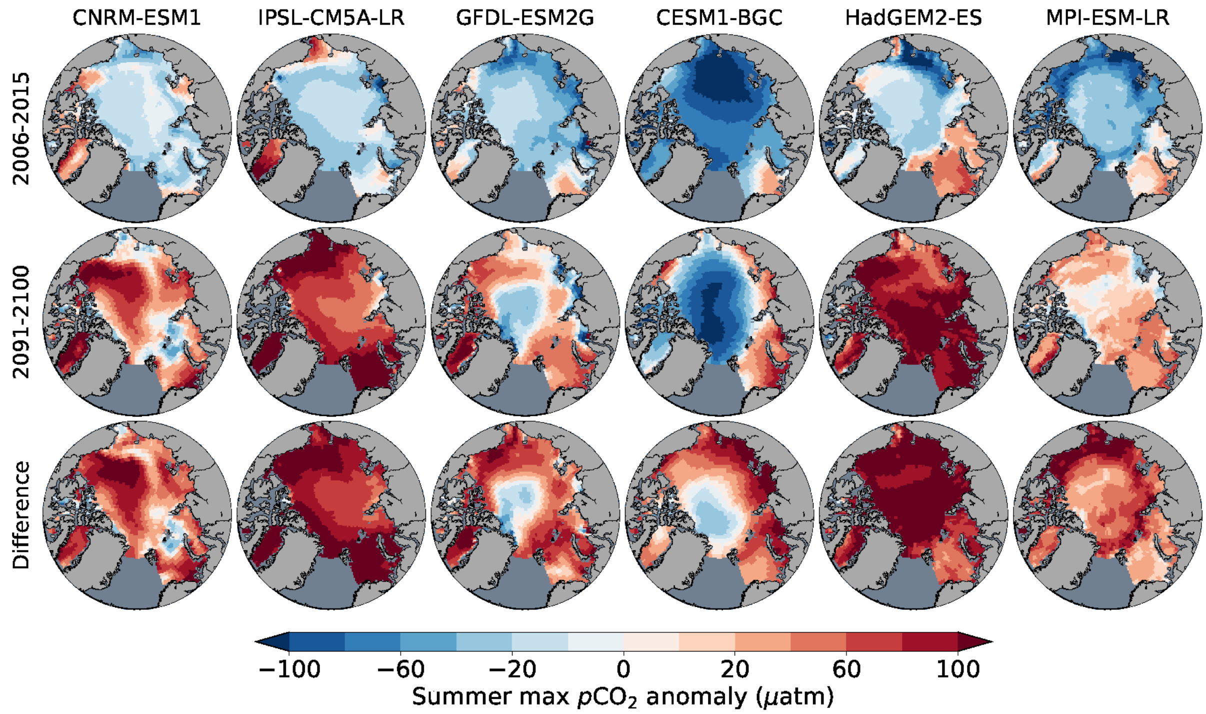

Supplementary Fig. 6 | **The sign of summer pCO_2' is reversed in the Arctic shelf seas in all CMIP5 models under RCP8.5.** The maximum from the summer monthly means (JAS) of pCO_2' is shown for 6 CMIP5 models under RCP8.5, given as decadal averages over 2006–2015 (top), 2091–2100 (middle), and their difference (bottom). Only one member of each pair of CMIP5 models from IPSL, GFDL, and MPI is shown because of similarities.

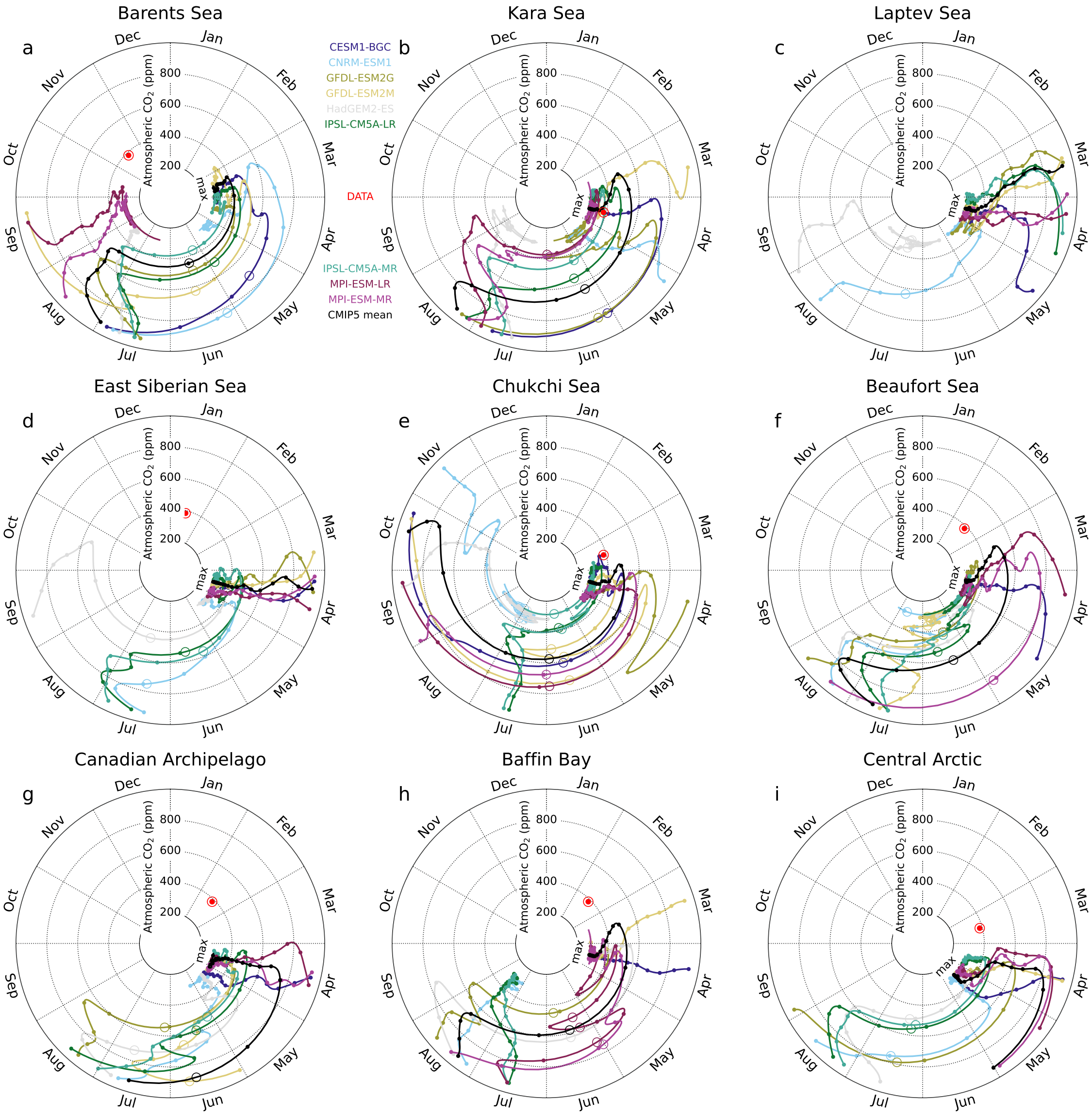

Supplementary Fig. 7 | **CMIP5 models exhibit crossover for $p\mathrm{CO}_2'$ sooner for shelf seas affected by Atlantic and Pacific inflow.** Evolution in seasonal timing of the annual high in surface ocean $p\mathrm{CO}_2'$ for the CMIP5 models under RCP8.5 averaged over **a**, Barents Sea, **b**, Kara Sea, **c**, Laptev Sea, **d**, East Siberian Sea, **e**, Chukchi Sea, **f**, Beaufort Sea, **g**, Canadian Archipelago, **h**, Baffin Bay, and **i**, Central Arctic. Averages include only the parts of the regions where the bathymetry is <500 m except for the Central Arctic (>500 m). Plot characteristics are as in Fig. 3.

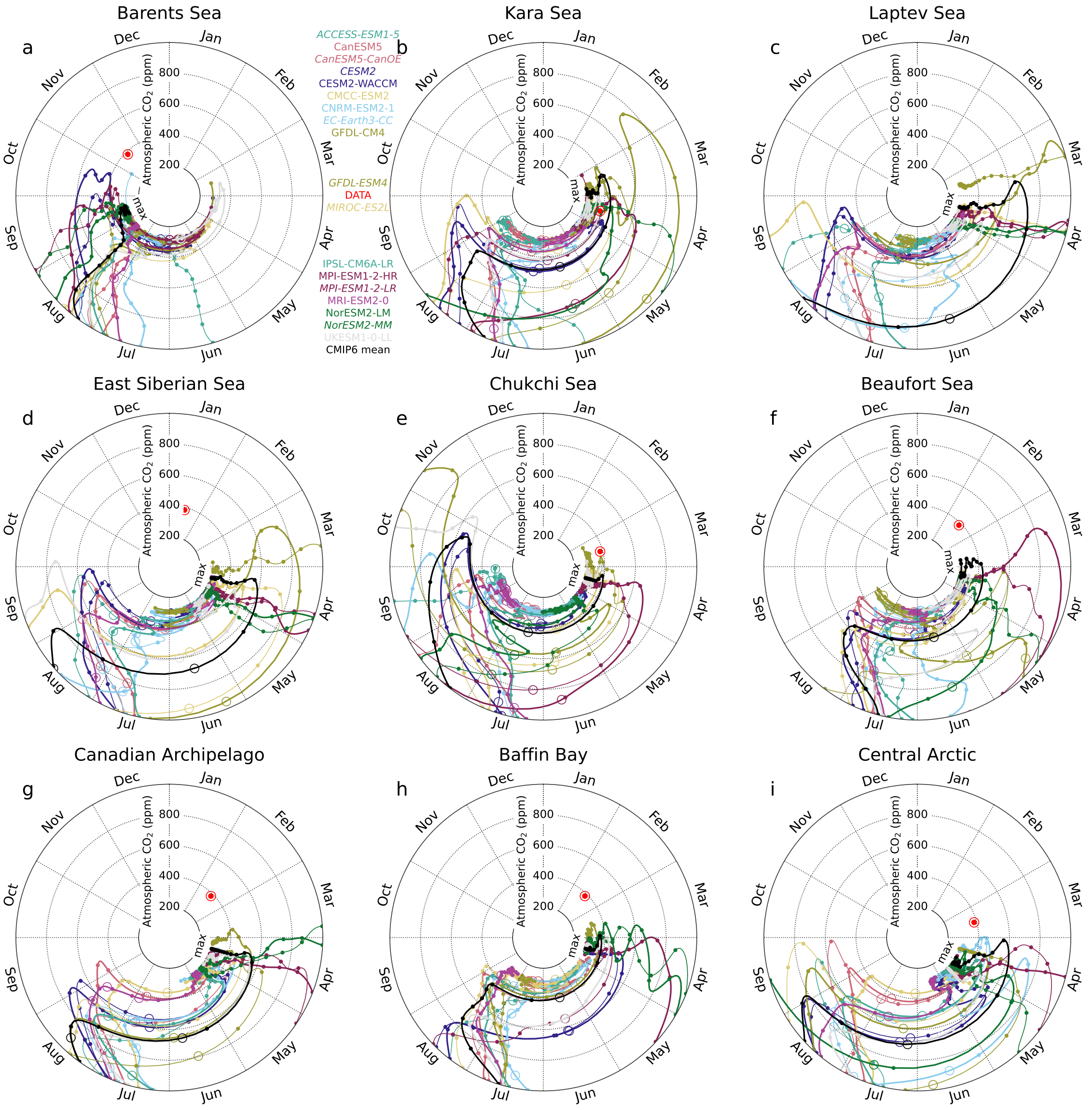

Supplementary Fig. 8 | **CMIP6 models exhibit crossover for pCO_2' sooner for shelf seas affected by Atlantic and Pacific inflow.** Evolution in seasonal timing of the annual high in surface ocean pCO_2' for the CMIP6 models under SSP5-8.5 averaged over **a**, Barents Sea, **b**, Kara Sea, **c**, Laptev Sea, **d**, East Siberian Sea, **e**, Chukchi Sea, **f**, Beaufort Sea, **g**, Canadian Archipelago, **h**, Baffin Bay, and **i**, Central Arctic. Averages include only the parts of the regions where the bathymetry is <500 m except for the Central Arctic (>500 m). Plot characteristics are as in Fig. 3.

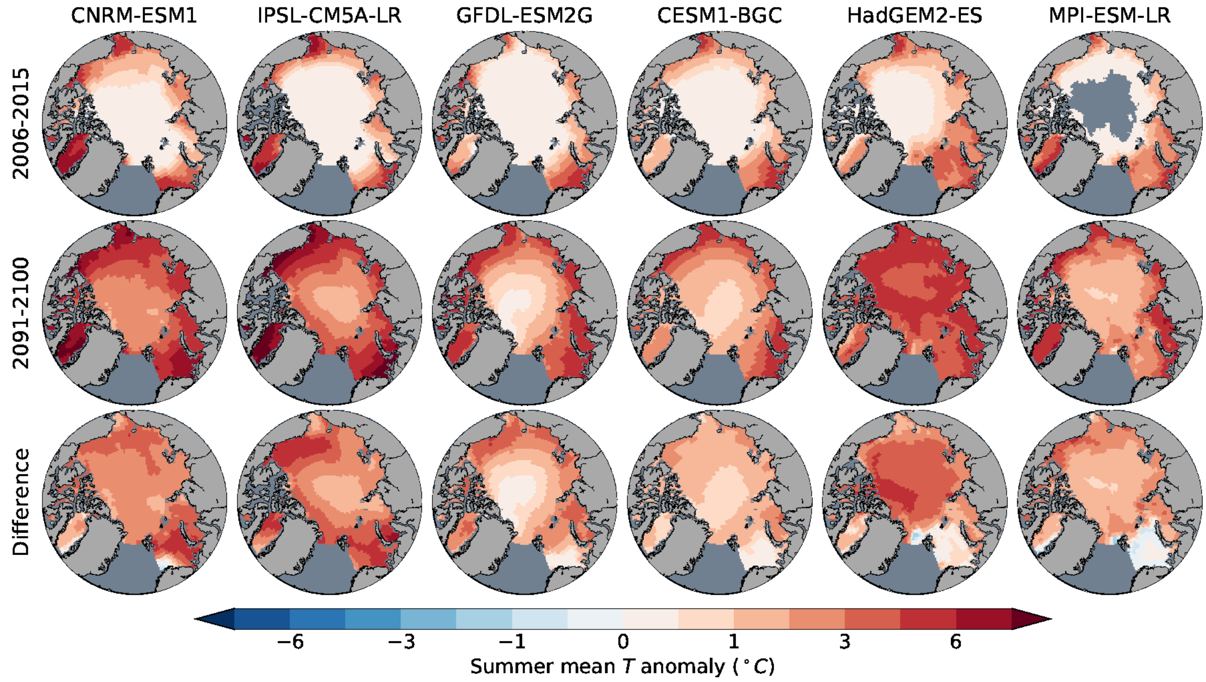

Supplementary Fig. 9 | **All CMIP5 models simulate enhanced summer warming of the Arctic shelf seas under RCP8.5.** Arctic maps of the summer mean anomaly for temperature (summer mean minus the annual mean) are shown for decadal averages over 2006–2015 (top), 2091–2100 (middle), and their difference (bottom).

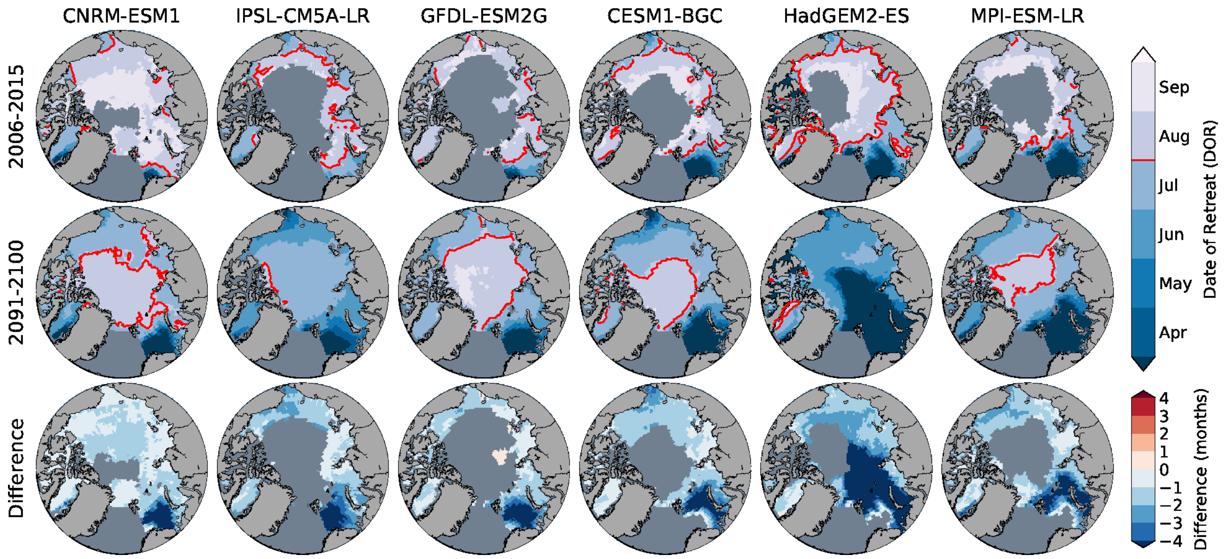

Supplementary Fig. 10 | **Models with earlier date of retreat (DOR) of sea ice tend to be warmer and have more positive $p\mathrm{CO}_2'$.** The DOR is calculated for the CMIP5 models under RCP8.5 as the month when the monthly mean sea ice concentration first falls below 0.15. The DOR is shown as decadal averages for 2006–2015 (top) and 2091–2100 (middle), while the difference in months between those periods is also given (bottom). The red contour line indicates the Late Summer Transition, occurring on 1 August, after which sea-ice retreat no longer results in substantially warmer surface waters because the net air-to-sea heat flux has become too weak[8]. Gray areas in the interior of the Arctic domain indicate regions where sea-ice concentration never falls below 0.15, a condition found in each model during 2006–2015, but not 2091–2100.

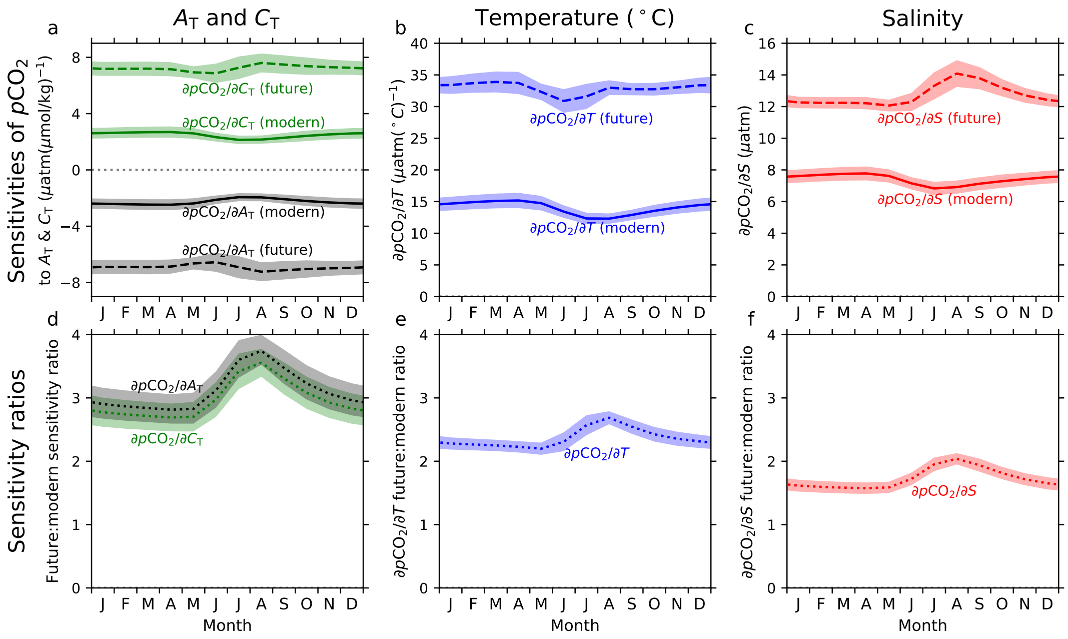

Supplementary Fig. 11 | **Sensitivities of pCO_2 to temperature increase but cannot explain the reversal of summer pCO_2'. a–c**, Sensitivities of pCO_2 to its drivers were computed for the CMIP5 models under RCP8.5 and are averaged here over Arctic domain for the modern (2006–2015, solid) and future (2091–2100, dashed) climatologies. Shown are sensitivities of pCO_2 to (**a**) A_T and C_T, (**b**) T', and (**c**) S'. **d–f**, The future-to-modern ratio of those sensitivities. Lines are for the CMIP5 mean and shading represents uncertainty (±1 s.d., n=9).

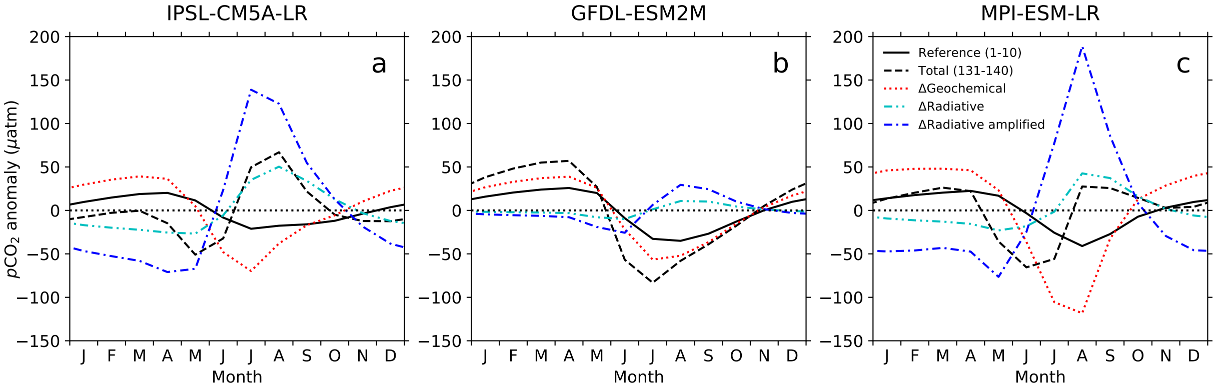

Supplementary Fig. 12 | **The idealized separation indicates that the amplified radiative effect dominates, corroborating the climate-CO_2 Taylor separation.** Shown are monthly anomalies in surface ocean $p$$CO_2$ from idealized simulations of the three CMIP5 models, which provided that output. Arctic-domain averages are plotted from the 140-year *1pctCO2* experiment for the first decade (Reference, black solid) and the final decade (Total, black dashes). Results from the other two experiments are given as perturbations, i.e., the change between the first and final decades of *esmFixClim1* (Geochemical, red dots) and *esmFdbk1* (Radiative, cyan dash-dot-dot). Panels also indicate the amplified Radiative effect (blue dash-dot) implied by the difference between the final decades of the *1pctCO2* and *esmFixClim1* experiments. That nonlinearity is the gap between the blue and cyan curves, i.e., the extent to which the sum of the reference (black solid), geochemical (red), and radiative (cyan) curves does not match the total curve (black dashed). Results from the GFDL-ESM2M model are nearly linear, but its atmospheric CO_2 was only doubled; in the other two models, it was quadrupled. Compare with the climate-CO_2 Taylor separation (Extended Data Fig. 5).

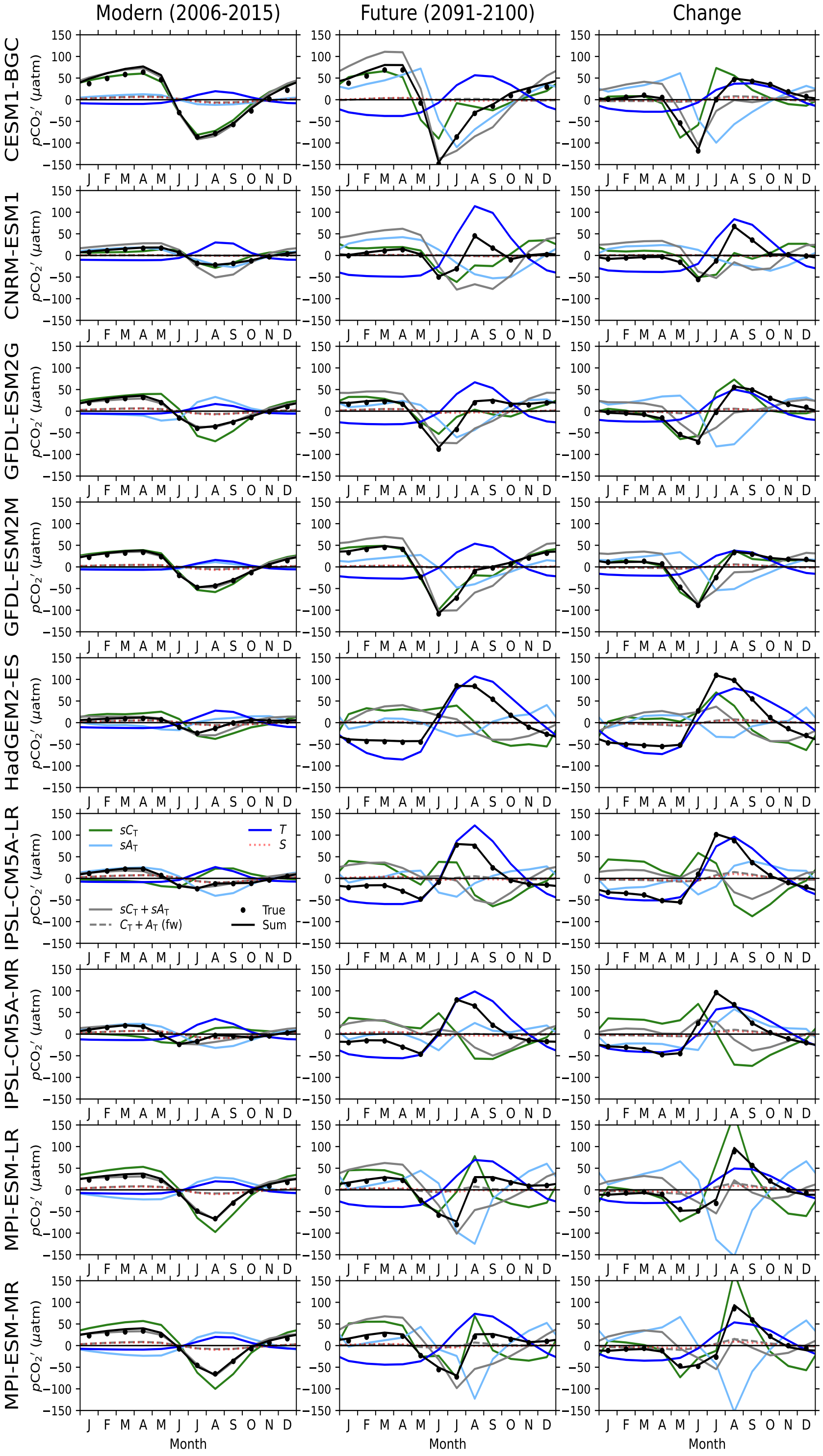

Supplementary Fig. 13 | **Future thermally driven variability dominates the change in summer pCO$_2$′, sharpening the annual minimum in all CMIP5 models under RCP8.5.** Results are from the freshwater Taylor expansion (equation 4).

Supplementary Table 1 | CMIP5 models used in this study.

Model	Institute
	CMIP5
CESM1-BGC[70,71]	National Center for Atmospheric Research, USA (NSF-DOE-NCAR)
CNRM-ESM1[72]	Centre National de Recherches Métórologiques, France (CNRM)
GFDL-ESM2G[73]	NOAA Geophysical Fluid Dynamics Laboratory, USA (NOAA-GFDL)
GFDL-ESM2M[73]	
HadGEM2-ES[74]	Met Office Hadley Centre, UK (MOHC)
IPSL-CM5A-LR[75]	Institut Pierre Simon Laplace, France (IPSL)
IPSL-CM5A-MR[75]	
MPI-ESM-LR[76]	Max-Planck-Institut für Meteorologie, Germany (MPI-M)
MPI-ESM-LR[76]	
	CMIP6
ACCESS-ESM1-5[77]	Commonwealth Scientific and Industrial Research Organisation, Australia (CSIRO)
CESM2[78]	National Center for Atmospheric Research, USA (NCAR)
CESM2-WACCM[78]	
CMCC-ESM2[79]	Fondazione Centro Euro-Mediterraneo sui Cambiamenti Climatici, Italy (CMCC)
CNRM-ESM2-1[80]	Centre National de Recherches Métórologiques, France (CNRM)
CanESM5[81]	Canadian Centre for Climate Modelling and Analysis, Canada (CCCMa)
CanESM5-CanOE[81]	
EC-Earth3-CC[82]	EC-Earth-Consortium, EU
GFDL-CM4[83,84]	NOAA Geophysical Fluid Dynamics Laboratory, USA (NOAA-GFDL)
GFDL-ESM4[85,86]	
IPSL-CM6A-LR[87]	Institut Pierre Simon Laplace, France (IPSL)
MIROC-ES2L[88]	Japan Agency for Marine-Earth Science and Technology et al., Japan (MIROC)
MPI-ESM1-2-HR[89,90]	Max Planck Institute for Meteorology, Germany (MPI)
MPI-ESM1-2-LR[89,90]	
MRI-ESM2-0[91]	Meteorological Research Institute, Japan (MRI)
NorESM2-LM[92]	NorESM Climate modeling Consortium, MET-Norway (NCC)
NorESM2-MM[92]	
UKESM1-0-LL[93]	Met Office Hadley Centre, UK (MOHC)

Supplementary Table 2 | Arctic domain pCO_2 timing and thermal metrics for CMIP models.

Model	Future timing of ocean pCO_2				$T^{max\ddagger}$ (°C)		
	min	max	atmospheric xCO_2 at Crossover¶ (ppm)	ECS§ (°C)	modern*	future*	change†
CMIP5 (RCP8.5)							
GFDL-ESM2M	Jun	Mar		2.4	0.1	3.1	3.0
CESM1-BGC	Jun	Apr		2.9	-0.2	3.1	3.3
GFDL-ESM2G	Jun	Mar,Sep	Converge‖	2.4	-0.2	3.4	3.7
MPI-ESM-LR	Jun	Apr,Aug	Converge	3.6	0.2	4.3	4.1
MPI-ESM-MR	Jun	Mar,Sep	923	3.5	0.2	4.5	4.2
CNRM-ESM1	Jun	Apr,Aug	744	3.1	1.3	6.4	5.1
IPSL-CM5A-MR	May	Jul	461	4.0	0.8	6.4	5.6
IPSL-CM5A-LR	May	Jul	536	4.1	0.2	6.5	6.2
HadGEM2-ES	May	Jul	400	4.6	0.7	8.4	7.6
CMIP5 mean	Jun	Aug	827	3.4	0.3	5.1	4.8
1 SD				0.7	0.5	1.8	1.4
CMIP6 (SSP5-8.5)							
NorESM2-MM	Jul	Apr		2.5	-0.4	3.2	3.6
GFDL-ESM4	Jun	Aug	956	2.6	0.8	4.9	4.1
NorESM2-LM	Jun	Sep	887	2.6	0.2	4.5	4.3
MPI-ESM1-2-LR	Jul	Sep	959	2.8	0.2	4.7	4.5
MPI-ESM1-2-HR	Jun	Apr	Converge	3.0	-0.1	4.7	4.7
MRI-ESM2-0	Jul	Jul	447	3.1	1.4	6.9	5.5
CESM2-WACCM	Mar	Jul	524	4.7	-0.2	5.6	5.7
CESM2	Apr	Jul	478	5.2	0.2	6.2	6.0
MIROC-ES2L	Jun	Aug	683	2.7	0.0	6.5	6.5
ACCESS-ESM1-5	Mar	Jul	449	3.9	1.5	8.2	6.6
GFDL-CM4	May	Aug	717	3.9	0.3	7.3	7.0
CMCC-ESM2	May	Aug	446	3.6	3.3	10.9	7.6
CanESM5	Feb	Jul	365	5.6	0.4	10.1	9.7
CNRM-ESM2-1	Apr	Jul	694	4.8	2.3	12.3	10.0
CanESM5-CanOE	Feb	Jul	399	5.6	0.2	10.5	10.3
EC-Earth3-CC	May	Jul	608	4.3	2.1	13.8	11.7
UKESM1-0-LL	Apr	Aug	579	5.4	0.1	12.4	12.3
IPSL-CM6A-LR	Aug	Jul	543	4.6	2.7	15.9	13.2
CMIP6 mean	May	Aug	571	3.9	0.8	8.3	7.4
1 SD				1.1	1.1	3.6	3.0

*modern and future are climatological averages for 1996–2005 and 2091–2100, respectively

†change is the difference between the modern and future climatologies

‡T^{max} indicates the maximum temperature (not debiased) of the 12 monthly means, which occurs in summer

§ECS is the equilibrium climate sensitivity[77,92,94–96]

¶Crossover is the atmospheric xCO_2 level at which the annual high and low in ocean pCO_2' switch chronological order

‖Converge indicates that the months of the annual high and low in pCO_2' grow closer but there is no crossover

Supplementary Table 3 | Additional change in summer and winter means (%) relative to the annual mean change during this century for the CMIP5 models (mean ± 1 s.d., n=9) under RCP8.5.

	Summer mean[a]			Winter mean[a]		
	Total	ΔSensitivities[b]	ΔAnomalies*[c]	Total	ΔSensitivities[b]	ΔAnomalies*[c]
pCO$_2$	9 ± 4	-11 ± 9	20 ± 7	-3 ± 4	6 ± 4	-9 ± 2
$[CO_2]$	-7 ± 3	-14 ± 7	7 ± 8	8 ± 1	9 ± 3	-2 ± 3
$[HCO_3^-]$	88 ± 92	18 ± 20	69 ± 74	-49 ± 58	-11 ± 10	-38 ± 50
$[H^+]$	7 ± 4	-8 ± 8	15 ± 6	-2 ± 3	5 ± 4	-6 ± 2
$[CO_3^{2-}]$	4 ± 8	7 ± 3	-2 ± 5	0 ± 4	-4 ± 2	4 ± 3

[a]Percent additional change in the seasonal mean relative to the annual-mean change between 2006–2015 to 2091–2100
[b]From changes in only the sensitivities (geochemical effect)
[c]From changes in the driver anomalies (radiative effect) and the synergy with the changes in sensitivities

68. Bakker, D. C. E. *et al.* A multi-decade record of high-quality fCO2 data in version 3 of the Surface Ocean CO2 Atlas (SOCAT). *Earth Syst. Sci. Data* **8,** 383–413 (2016).

69. Gleckler, P. J., Taylor, K. E. & Doutriaux, C. Performance metrics for climate models. *J. Geophys. Res.* **113,** D06104 (2008).

70. Gent, P. R. *et al.* The Community Climate System Model Version 4. *J. Clim.* **24,** 4973–4991 (2011).

71. Lindsay, K. *et al.* Preindustrial-Control and Twentieth-Century Carbon Cycle Experiments with the Earth System Model CESM1(BGC). *J. Clim.* **27,** 8981–9005 (2014).

72. Voldoire, A. *et al.* The CNRM-CM5.1 global climate model: description and basic evaluation. *Clim. Dyn.* **40,** 2091–2121 (2012).

73. Dunne, J. P. *et al.* GFDL's ESM2 Global Coupled Climate-Carbon Earth System Models. Part I: Physical Formulation and Baseline Simulation Characteristics. *J. Clim.* **25,** 6646–6665 (2012).

74. Collins, W. J. *et al.* Development and evaluation of an Earth-System model – HadGEM2. *Geosci. Model Dev.* **4,** 1051–1075 (2011).

75. Dufresne, J.-L. *et al.* Climate change projections using the IPSL-CM5 Earth System Model: from CMIP3 to CMIP5. *Clim. Dyn.* **40,** 2123–2165 (2013).

76. Giorgetta, M. A. *et al.* Climate and carbon cycle changes from 1850 to 2100 in MPI-ESM simulations for the Coupled Model Intercomparison Project phase 5. *J. Adv. Model. Earth Syst.* **5,** 572–597 (2013).

77. Ziehn, T. *et al.* The Australian Earth System Model: ACCESS-ESM1.5. *J. South. Hemisph. Earth Syst. Sci.* **70,** 193–214 (2020).

78. Danabasoglu, G. *et al.* The Community Earth System Model Version 2 (CESM2). *J. Adv. Model. Earth Syst.* **12,** e2019MS001916 (2020).

79. Cherchi, A. *et al.* Global mean climate and main patterns of variability in the CMCC-CM2 coupled model. *J. Adv. Model. Earth Syst.* **11,** 185–209 (2019).

80. Séférian, R. *et al.* Evaluation of CNRM Earth System Model, CNRM-ESM2-1: Role of Earth System Processes in Present-Day and Future Climate. *J. Adv. Model. Earth Syst.* **11,** 4182–4227 (2019).

81. Swart, N. C. *et al.* The Canadian Earth System Model version 5 (CanESM5.0.3). *Geosci. Model Dev.* **12,** 4823–4873 (2019).

82. Döscher, R. *et al.* The EC-Earth3 Earth system model for the Coupled Model Intercomparison Project 6. *Geoscientific Model Development* **15,** 2973–3020 (2022).

83. Held, I. M. *et al.* Structure and Performance of GFDL's CM4.0 Climate Model. *J. Adv. Model. Earth Syst.* **11,** 3691–3727 (2019).

84. Dunne, J. P. *et al.* The GFDL Earth System Model Version 4.1 (GFDL-ESM 4.1): Overall Coupled Model Description and Simulation Characteristics. *J. Adv. Model. Earth Syst.* **12,** e2019MS00201 (2020).

85. Dunne, J. P. *et al.* Simple Global Ocean Biogeochemistry With Light, Iron, Nutrients and Gas Version 2 (BLINGv2): Model Description and Simulation Characteristics in GFDL's CM4.0. *J. Adv. Model. Earth Syst.* **12,** e2019MS002008 (2020).

86. Stock, C. A. *et al.* Ocean Biogeochemistry in GFDL's Earth System Model 4.1 and Its Response to Increasing Atmospheric CO2. *J. Adv. Model. Earth Syst.* **12,** e2019MS002043 (2020).

87. Boucher, O. *et al.* Presentation and Evaluation of the IPSL-CM6A-LR Climate Model. *J. Adv. Model. Earth Syst.* **12,** e2019MS00201 (2020).

88. Hajima, T. *et al.* Development of the MIROC-ES2L Earth system model and the evaluation of biogeochemical processes and feedbacks. *Geosci. Model Dev.* **13,** 2197–2244 (2020).

89. Müller, W. A. *et al.* A higher-resolution version of the Max Planck Institute Earth System Model (MPI-ESM1.2-HR). *J. Adv. Model. Earth Syst.* **10,** 1383–1413 (2018).

90. Mauritsen, T. *et al.* Developments in the MPI-M Earth System Model version 1.2 (MPI-ESM1.2) and Its Response to Increasing CO2. *J. Adv. Model. Earth Syst.* **11,** 998–1038 (2019).

91. Yukimoto, S. *et al.* The Meteorological Research Institute Earth System Model Version 2.0, MRI-ESM2.0: Description and Basic Evaluation of the Physical Component. *J. Meteorolog. Soc. Japan. Ser. II* **97,** 931–965 (2019).

92. Seland, Ø. *et al.* Overview of the Norwegian Earth System Model (NorESM2) and key climate response of CMIP6 DECK, historical, and scenario simulations. *Geosci. Model Dev.* **13,** 6165–6200 (2020).

93. Sellar, A. A. *et al.* UKESM1: Description and Evaluation of the U.K. Earth System Model. *J. Adv. Model. Earth Syst.* **11,** 4513–4558 (2019).

94. Meehl, G. A. *et al.* Context for interpreting equilibrium climate sensitivity and transient climate response from the CMIP6 Earth system models. *Sci. Adv.* **6,** eaba1981 (2020).

95. Flynn, C. M. & Mauritsen, T. On the climate sensitivity and historical warming evolution in recent coupled model ensembles. *Atmos. Chem. Phys.* **20,** 7829–7842 (2020).

96. Nohara, D. *et al.* Examination of a climate stabilization pathway via zero-emissions using Earth system models. *Environ. Res. Lett.* **10,** 095005 (2015).
