# Supplementary material for: Arctic Ocean annual high in pCO2 could shift from winter to summer
Source: Nature. 2022 Oct 5;610(7930):94–100. doi: 10.1038/s41586-022-05205-y (PMC9534769; doi:10.1038/s41586-022-05205-y)
Supplement: Supplementary file 2 — Peer Review File [file 41586_2022_5205_MOESM2_ESM.pdf]

**Peer Review File**

**Manuscript Title:** Arctic Ocean annual high in $pCO_2$ could shift from winter to summer

## Reviewer Comments & Author Rebuttals

### Reviewer Reports on the Initial Version:

Referees' comments:

Referee #1 (Remarks to the Author):

Review of the manuscript entitled “Projected disruption in seasonal timing of the Arctic Ocean CO2 system” by Orr et al.

Summary:

The manuscript of Orr et al., (2022) assessed the monthly variations in sea surface pCO2 and related carbonate chemistry and their potential future changes in the Arctic Ocean via CIMP5 and CIMP6 results. The authors used Taylor-series expansions and model idealized experiments to assess and distinguish the possible drivers. One most interesting and important finding is that the current summer low pCO2 anomaly will split into a spring-summer minimum and a summer maximum in the future, which may worsen summer surface ocean acidification in the Arctic Ocean. Although recent observational studies have noticed that summer pCO2 rapidly increase (possibly splitting from spring-summer low pCO2) in the ice-free southern Canada Basin and the Beaufort Sea in the recent decades due to sea ice retreat, warming and air-sea CO2 uptake (DeGrandpre et al., 2020; Ouyang et al., 2020), this phenomenon have not been fully understood and assessed over the basin-wide Arctic Ocean, even less is known about the possible future change. This manuscript provides valuable insights into such changes in timing and seasonality of the surface pCO2 anomaly and its evolution from modern Arctic Ocean to the future Arctic Ocean under high-end emissions scenario. In addition, the authors nicely discussed the different responses of other associated carbonate parameters to the further climate scenario and the possible impacts on marine organisms. This manuscript must be of great interest to researchers in the disciplines of biogeochemical cycles, marine biology, and ocean acidification driven by climate change.

In general, I find this manuscript is very well written and interesting to read. The main text is supported by several well-presented figures. The authors are careful, clear, and concise as they explain the methods. I believe this work will be an important contribution and worthy of publication in a high-impact journal like Nature. Here, I have some concerns and questions for the author to consider prior to publication.

Reference:
DeGrandpre, M., Evans, W., Timmermans, M. L., Krishfield, R., Williams, B., & Steele, M. (2020). Changes in the arctic ocean carbon cycle with diminishing ice cover. Geophysical research letters, 47(12), e2020GL088051.
Ouyang, Z., Qi, D., Chen, L., Takahashi, T., Zhong, W., DeGrandpre, M. D., ... & Cai, W. J. (2020). Sea-

nature portfolio

ice loss amplifies summertime decadal CO2 increase in the western Arctic Ocean. Nature Climate Change, 10(7), 678-684.

Major comments:

Timing of crossover for pCO2 anomaly vs. summer ice-free Arctic Ocean
In the manuscript, the author mainly compared monthly pCO2 anomaly in two decades (i.e., 1996-2005 vs. 2091-2100), and attributed newly appeared summer high pCO2 (sign reversal) to large summer surface warming driven by earlier retreat of sea ice. Although the authors explained the timing of crossover of pCO2 anomaly by giving the atmospheric xCO2 concentration at crossover, it is not clear how the splitting and inversion of summer low pCO2 anomaly is related to future sea ice extend decline. I am curious if the earliest splitting and inversion will occur when the Arctic Ocean will become ice-free or nearly ice-free in summer in 2030s (Wang and Overland, 2012; Overland & Wang, 2013). As sea ice suppresses surface warming and pCO2 increase via air-sea exchange, it is likely that the mean timing of splitting and inversion of summer low pCO2 will occur as early as 2030s because surface warming tends to accelerate in an ice-free Arctic. Although the spatial variation (shelf vs. basin) may induce large uncertainty, it is still worthy to discuss. If the timing of these two processes (timing of crossover for pCO2 anomaly vs. summer ice-free Arctic Ocean) are strongly related to each other, it may provide a possible mechanism to further support the conclusion in this study that thermal effect results in summer high pCO2 and dominate pCO2 anomaly, even stronger when summer sea ice disappears. In addition, reporting the earliest timing of summer pCO2 anomaly splitting may remind the readers that the impact of ocean acidification due to summer high pCO2 may occur much earlier than the decades of 2091-2100.

Reference:
Wang, M., & Overland, J. E. (2012). A sea ice free summer Arctic within 30 years: An update from CMIP5 models. Geophysical Research Letters, 39(18).
Overland, J. E., & Wang, M. (2013). When will the summer Arctic be nearly sea ice free?. Geophysical Research Letters, 40(10), 2097-2101.

Possible biological control
As biological drawdown of CO2 is a crucial process controlling seasonal variation in sea surface pCO2, I am wondering how the models handle the possible changes in future biological process in both spatial variation and seasonal magnitudes. Several recent studies have examined temporal and spatial variations in Arctic primary production, as well as the timing of phytoplankton bloom in recent decades in the Arctic Ocean (Song et al., 2021; Lewis et al., 2020; Ardyna et al., 2014). Although it is uncertain that the future changes in biological process will follow the pattern and mechanism which were found in these studies, it is worthy and helpful to have a discussion on how the possible change in the future biological processes may alter the future pCO2 anomaly. For example, the fall bloom on the Arctic shelve may damper the summer high pCO2 anomaly. It may also weaken the finding in this study that “The tendency for pCO2 to reverse sign in summer during this century is particularly pronounced in the Arctic shelf seas (Fig. 4).”

Reference:
Song, H., Ji, R., Jin, M., Li, Y., Feng, Z., Varpe, Ø., & Davis, C. S. (2021). Strong and regionally distinct

links between ice-retreat timing and phytoplankton production in the Arctic Ocean. Limnology and Oceanography, 66(6), 2498-2508.
Lewis, K. M., Van Dijken, G. L., & Arrigo, K. R. (2020). Changes in phytoplankton concentration now drive increased Arctic Ocean primary production. Science, 369(6500), 198-202.
Ardyna, M., Babin, M., Gosselin, M., Devred, E., Rainville, L., & Tremblay, J. É. (2014). Recent Arctic Ocean sea ice loss triggers novel fall phytoplankton blooms. Geophysical Research Letters, 41(17), 6207-6212.

Negative $pCO_2$ anomaly in winter under SSP5-8.5 (CMIP6)
Based on current observations, winter sea surface $pCO_2$ is usually close to the atmospheric $CO_2$, even higher in some location due to sea ice formation and possible respiration beneath the ice. I am surprised to see that mean $pCO_2$ anomaly under SSP5-8.5 scenario (CMIP6) for 2091-2100 becomes negative in winter and spring (Extended data Fig. 1b), which is not consistent with the expectation that $pCO_2$ in winter should be above the annual mean or at least close to the annual mean. Among all scenarios in Extended data Fig. 2, the case under SSP5-8.5 (CMIP6) is the only one that mean $pCO_2$ anomaly become negative in winter. Why? Needs more explanation. If it is confirmed to be the case, this result may have an important implication that the Arctic Ocean has a tendency to transform to from neutral state in modern to a weak $CO_2$ sink in the future winter.

Minor comments:
Line 67-70: Recent compilation studies of sea surface $pCO_2$ have already showed that summer high $pCO_2$ appeared (splitting from low summer $pCO_2$) in the southern Canada Basin and the Beaufort Sea in the recent decades (DeGrandpre et al., 2020; Ouyang et al., 2020), which was associated with earlier ice retreat and greater local warming and limited biological $CO_2$ drawdown.

Reference:
DeGrandpre, M., Evans, W., Timmermans, M. L., Krishfield, R., Williams, B., & Steele, M. (2020). Changes in the arctic ocean carbon cycle with diminishing ice cover. Geophysical research letters, 47(12), e2020GL088051.
Ouyang, Z., Qi, D., Chen, L., Takahashi, T., Zhong, W., DeGrandpre, M. D., ... & Cai, W. J. (2020). Sea-ice loss amplifies summertime decadal CO2 increase in the western Arctic Ocean. Nature Climate Change, 10(7), 678-684.

Line 94: delete one “and” in the sentence

Line 95: It is better to briefly explain what is “equilibrium climate sensitivity (ESC)” and its implication. So, the readers can more easily follow the text flow.

Line 97: How to explain the non-thermal component of summer $pCO_2$ anomaly decreases in the shelves?

Line 104: Not all model project warming across the Arctic shelf sea (supplementary Fig. 6), e.g., cooling in the Barents Sea in MPI-ESM-LR.

Line 106: Does the statement of “after which the net air-to-sea heat flux becomes so low that it fails

to warm surface waters" will still hold true in the 2091-2100 as the sea ice will retreat earlier and warming may extend beyond 1Aug?

Line 147: Supplementary Table 2?

Line 171: What is the possible cause or process driving the salinity normalized AT term to become more important during 2091-2100?

Line 201: …for that season's mean and 23±8% for its extreme (Supplementary Table 3). According to Supplementary Table 3, "23±8%" should be "21±8%". Please double check.

Line 474: Delete one "variables"

Line 550: I am confused about the description of equation terms. When you mentioned "the first right-hand side term in equ (3), are you intending to say "the first left-hand side term"?

Figures:
Fig.2: … Extended Data Fig. 1 shows analogous results from CMIP6 (SSP5-8.5). Add a period. Also, need to explain what is the shading.

Fig. 5: The sentence, "agreement (±1σ, 9 models) but is omitted for the two black curves in the top panel, being already shown in Fig. 2.", seems does not work.
Also need to explain what is the shading (uncertainty?)

Extended Data Fig. 1: Need to explain what is the shading.

Extended Data Fig. 6: Need to explain what is "True" dots in the plots.

Referee #2 (Remarks to the Author):

The manuscript presents a pertinent and scientifically interesting interpretation of future projected changes in the seasonality of the Arctic surface ocean carbon cycle over the 21st century with strong emissions. I think this novel work should be of interest to the broad scientific community and attain the standard rehired for Nature, following text re-organization and streamlining as identified below. The methods are valid, the conclusions are robust, and with minor changes the abstract, summary, and conclusions convey the important points effectively.

The comments are organized below by major and minor points.

Major Points:

First and foremost, I believe that the scientific point of timing emphasized in Fig. 3 and Extended

Data Fig. 3 is intriguing. In particular for Extended FIg. 3b and 3d the relatively abrupt (over 20-30 years) transition for the month of maximum pCO2 shows that (albeit often at different CO2 levels) most models exhibit a dramatic shift. I would suggest combining Fig. 3 and Extended Data FIg. 3 to emphasize this in the main text, if this can be allowed with space requirements. The details of the responses of the individual models is sufficiently important to warrant this. But it is also important that the authors return to this question in their interpretation at the end of the paper. Why is it that this transition is triggered at disparate atmospheric CO2 levels for the different models? Much of the analysis here focuses on the phasing and anomalies, but it would be valuable for the authors to connect this with the warming and ice retreat signals more directly, and to identify whether or not there are specific thresholds that trigger this rapid transition.

One a related note, on a first reading of the manuscript, I was also perplexed by Fig. 2b, where at first glance it appears that there is wild model uncertainty in the forced changes in the seasonal phasing of pCO2 anomalies. From this figure alone I wasn't satisfied as a reader that calculating a multi-model ensemble was reasonable for this story, but it was more in the details of the Extended Figures that the authors managed to convince me that this was a reasonable choice to evaluate an ensemble mean rather than looking at two different clusters or groupings for the fields shown in Fig. 2b. I would recommend that the authors move Fig. 1 to the Supplementary Materials, since I think it is confusing to present this ensemble mean view without first showing the behavior of the individual underlying models.

In characterizing what is special about the Arctic, I believe that in their discussion the authors should also describe (and reference) what is known about the seasonality component of Arctic amplification as a physical climate phenomenon. This would not require more than a few sentences to make a statement about this and then to connect this to the carbon story.

Last but not least with the main comments on content, I think that the final section on “Biological Impacts” cold be strengthened by up-fronting more specific information about species and ecosystems that are vulnerable. Given that the authors commented in the earlier part of the manuscript not the work of McNeill and Sasse, it would be helpful if they could comment a bit on the relative importance of hypercapnia, acificiation, etc. for organism and ecosystem vulnerabilities. The authors do an effective job of relating both chemical and biological mechanisms that could be important, but it would be greatly beneficial if this could be reshaped to be more directly accessible to a broader audience, including but not limited to ecologists. The authors emphasize pCO2 anomalies, but the absolute levels are also interesting and important.

More specific points:

In the Abstract, the authors mention the retreat of sea ice, but they don't state there that the changes in seasonality have an abruptness to them that is important. I would recommend mentioning this in the Abstract.

In the final sentence of the Abstract, again I wonder if hypercapnia should also be considered in this context.

Line 27: the study of Rodgers et al. (2008) should be referenced with respect to forced increases in pCO2 seasonality for the surface ocean.

Line 51: the authors might consider saying “newly revised” rather than “new”, unless there is something entirely new about the treatment of the Arctic, in which case this should be explicitly stated.

Lines 58-62: See my comments above, I think that Fig. 1 should be moved to the Supplementary Materials.

Lines 64-68: are the authors sure that both CO2 and NPP aren’t responding to the warming signal, insofar as sea ice retreat can impact light levels in the Arctic.

Line 160: I think that the section header “Term separation” is too technical, why not change this to be “Mechanistic Attribution” or “Underlying Mechanisms”?

Line 166: should be “salinity-normalized”

Line 222: As noted above, I think it would be much better to begin here with something about specific organisms/ecosystems that are vulnerable, and the relative importance of pCO2 directly acting as a stressor relative to other stressors (acidification and heating). The science here is fine, but the authors should really make this more accessible.

Fig. 2 caption: Need to explain grey shading

Fig. 3 caption: should state why no “minimum” is shown for NPP

Extended Data Fig. 3: should also mention for this figure that small sold circles mark the end of decades

Line 368 and line 372: would it make sense to use the nomenclature BGC and RAD to refer to the esmFixClim1 and esmFdbkl experiments?

Referee #3 (Remarks to the Author):

Review of “Projected disruption in seasonal timing of the Arctic Ocean CO2 system”

By Orr et al

Submitted to Nature

The main message of the paper is that the combined effects of ocean acidification and climate

change driven warming reverses the seasonal cycle of pCO2 and H+ in the Arctic, turning the summer low of pCO2 and H+ into a seasonal high. The authors use a suite of CMIP5 and 6 models, and a neural network data product, combined with Taylor expansions and a series of specific simulations to determine the effects of different drivers on the inorganic carbon system.

Overall I think this is a well-written, novel, timely paper and I really enjoyed reading it. Figures are clear and support the major findings. I have a few comments that are hopefully useful to the authors.

Title:
I usually do not comment on the title of a paper, but since I am very excited about this work I will give my two cents anyway:
I find that the title is not very clear and is understating what was found in this study. Currently, I don’t think the title is accessible to the broad Nature readership.
How about: “The combined effects of OA and climate change shift annual high of pCO2 from winter to summer.” Or something along these lines?

Main Text:
Again, to make it more accessible to the broader audience I would suggest adding a few sentences about the biological relevance of this finding right on page two, within the first paragraph.

p. 3. L 54-57
Sentence is hard to read. Split into two.

p. 3, L 58-60
This sentence is hard to read. Maybe get rid of “For the future” and exchange end of the century with “end of 2100”. Or split up sentence.

p. 4 L64 -65
I don’t see an earlier peak in NPP

p. 6 L113
“For a wider view” needs to be reworded. It is unclear what the authors are trying to say here.

P 12 L 222-223
Maybe simplify to "CaCO3 dissolution and production both depend on CO32"

P 13 L257:
This is a bit confusing. As it reads now it says to me: summer pCO2 was already extreme in the past, but in the future it will be 4 times as extreme." But the extreme used to happen in winter... Maybe rephrase?

P 13 L258-261
This is a very important message. Can you make this stronger? Maybe even use the term

multistressor, and a bit more info about current research on the impact of these two combined stressors? or even bring in some discussion on compound event?

P55 L476-478
This is confusing. Why CMIP5 CO2 system variable?

P57 L 506-507
If you debias max temperature doesn't it affect your resutls from the Taylor expansion?

Section on Freshwater Taylor series expansion:
It is not clear if the authors used a non-zero TA and DIC end member to salinity normalize these variables. If not I would encourage them to look into it. It makes a big difference for the Arctic. See Friis et al., 2003 GEOPHYSICAL RESEARCH LETTERS, VOL. 30, NO. 2, 1085, doi:10.1029/2002GL015898, 2003. If zero TA and DIC endmembers are used, it needs to be shown why it is ok.

Figure 2
Please define shaded area

Figure 3
Please define back dashed line

Overall comment regarding the use of CMIP5 and CMIP6: It might be useful to mention why simulations from both intercomparison projects were used. What do we learn from one vs. the other?

Reviewed by Claudine Hauri

**Author Rebuttals to Initial Comments:**

**Referee #1**:

*Summary:*

*The manuscript of Orr et al., (2022) assessed the monthly variations in sea surface pCO2 and related carbonate chemistry and their potential future changes in the Arctic Ocean via CIMP5 and CIMP6 results. The authors used Taylor-series expansions and model idealized experiments to assess and distinguish the possible drivers. One most interesting and important finding is that the current summer low pCO2 anomaly will split into a spring-summer minimum and a summer maximum in the future, which may worsen summer surface ocean acidification in the Arctic Ocean. Although recent observational studies have noticed that summer pCO2 rapidly increase (possibly splitting from spring-summer low pCO2) in the ice-free southern Canada Basin and the Beaufort Sea in the recent decades due to sea ice retreat, warming and air-sea CO2 uptake (DeGrandpre et al., 2020; Ouyang et al., 2020), this phenomenon have not been fully understood and assessed over the basin-wide Arctic Ocean, even less is known about the possible future change. This manuscript provides valuable insights into such changes in timing and seasonality of the surface pCO2 anomaly and its evolution from modern Arctic Ocean to the future Arctic Ocean under high-end emissions scenario. In addition, the authors nicely discussed the different responses of other associated carbonate parameters to the further climate scenario and the possible impacts on marine organisms. This manuscript must be of great interest to researchers in the disciplines of biogeochemical cycles, marine biology, and ocean acidification driven by climate change.*

*In general, I find this manuscript is very well written and interesting to read. The main text is supported by several well-presented figures. The authors are careful, clear, and concise as they explain the methods. I believe this work will be an important contribution and worthy of publication in a high-impact journal like Nature. Here, I have some concerns and questions for the author to consider prior to publication.*

These general comments are much appreciated. The two references that are mentioned above are now brought up in the revised manuscript as detailed in our response to the minor comments below.

*Reference:*
*DeGrandpre, M., Evans, W., Timmermans, M. L., Krishfield, R., Williams, B., & Steele, M. (2020). Changes in the arctic ocean carbon cycle with diminishing ice cover. Geophysical research letters, 47(12), e2020GL088051.*
*Ouyang, Z., Qi, D., Chen, L., Takahashi, T., Zhong, W., DeGrandpre, M. D., ... & Cai, W. J. (2020). Sea-ice loss amplifies summertime decadal CO2 increase in the western Arctic Ocean. Nature Climate Change, 10(7), 678-684.*

*Major comments:*

*Timing of crossover for pCO2 anomaly vs. summer ice-free Arctic Ocean*
*In the manuscript, the author mainly compared monthly pCO2 anomaly in two decades (i.e., 1996-2005 vs. 2091-2100), and attributed newly appeared summer high pCO2 (sign reversal) to large summer surface warming driven by earlier retreat of sea ice. Although the authors explained the timing of crossover of pCO2 anomaly by giving the atmospheric xCO2 concentration at crossover, it is not clear how the splitting and inversion of summer low pCO2 anomaly is related to future sea ice extend decline. I am curious if the earliest splitting and inversion will occur when the Arctic Ocean will become ice-free or nearly ice-free in summer in 2030s (Wang and Overland, 2012; Overland & Wang, 2013). As sea ice suppresses surface warming and pCO2 increase via air-sea exchange, it is likely that the mean timing of splitting and inversion of summer low pCO2 will occur as early as 2030s because surface warming tends to accelerate in an ice-free Arctic. Although the spatial variation (shelf vs. basin) may induce large uncertainty, it is still worthy to discuss. If the timing of these two processes (timing of crossover for pCO2 anomaly vs. summer ice-free Arctic Ocean) are strongly related to each other, it may provide a possible mechanism to further support the conclusion in this study that thermal effect results in summer high pCO2 and dominate pCO2 anomaly, even stronger when summer sea ice disappears. In addition, reporting the earliest timing of summer pCO2 anomaly splitting may remind the readers that the impact of ocean acidification due to summer high pCO2*



nature portfolio

*may occur much earlier than the decades of 2091-2100.*

In summary, Referee #1 would like us to clarify how the change in the seasonal cycle of $pCO_2$ is related to sea ice decline, would like to know if the earliest splitting and inversion will occur when the Arctic Ocean becomes nearly ice free, and would like us to report on that timing to emphasize that effects may occur well before the end of the century.

These remarks made us realize that our submitted manuscript was too vague about the evolution of the full seasonal cycle. We had showed the full seasonal cycle only at the beginning and end of this century (Figs. 1 and 2, line plots) along with the evolution of two metrics of the seasonal cycle, the annual high and low (Fig. 3, polar plots). That was insufficient to properly address the concerns raised by Referee #1. To remedy this problem, we now also show the evolution of the full seasonal cycle. For that, we have added two new panels (c and d) to Fig. 3, one for the CMIP5 mean and the other for the CMIP6 mean. They each show $pCO_2'$ in all 12 months with a separate line for climatological averages of 1861-1870 and each decade over this century. These 2 new panels reveal that unlike the abrupt change in the annual high, the change in summer $pCO_2'$ is gradual. So in the paragraph where Fig. 3 is introduced, we now end it with the following 2 sentences:

*Despite the abrupt transition in annual maximum $pCO_2'$ from winter to summer, the increase in summer $pCO_2'$ is a gradual evolution (Fig. 3c,d), the range of which seems to contain the different model behaviours seen in 2091–2100 (Fig. 2 and Extended Data Fig. 1).*

Furthermore, the gradual increase in summer $pCO_2'$ is from the growing thermal term as demonstrated quantitatively later in the manuscript. As the thermal term grows, it reaches the point where it outweighs the combined effects from all other terms (nonthermal) and summer $pCO_2'$ becomes positive (light blue curves in Fig. 3c,d). With further growth, the summer maximum surpasses the winter maximum and becomes the annual maximum (thick orange curve), at which point we see the abrupt transition shown in Fig. 3a,b. The growth in the thermal term generally begins before sea ice disappears for both the CMIP5 and CMIP6 means. But that is not the only factor, an important point that we now develop at the end of this section in a new paragraph:

*Yet warming from sea-ice retreat does not act alone on $pCO_2'$. Generally opposed in sign is the nonthermal contribution, which varies in models, e.g., due to different biogeochemical components. It is the balance of the nonthermal contribution with the gradually increasing thermal contribution that determines when summer $pCO_2'$ becomes positive and when it surpasses the winter maximum, both abrupt transitions. Models differ in $pCO_2'$ because nonthermal as well as thermal contributions differ, as suggested by their large differences in T' and NPP[5] (Fig. 2 and Extended Data Fig. 1). Although modelled NPP varies greatly and is imperfect, e.g., not capturing observation-based estimates of regional differences[22] and recent changes in phenology[23,24], and observations cannot tell us whether NPP will continue to increase[25] or decline[26] during this century, the use of many diverse models lends confidence that they would encompass the real ocean response. Weighing these contributions requires a quantitative framework.*

*Reference:*
*Wang, M., & Overland, J. E. (2012). A sea ice free summer Arctic within 30 years: An update from CMIP5 models. Geophysical Research Letters, 39(18).*
*Overland, J. E., & Wang, M. (2013). When will the summer Arctic be nearly sea ice free?. Geophysical Research Letters, 40(10), 2097-2101.*

*Possible biological control*
*As biological drawdown of CO2 is a crucial process controlling seasonal variation in sea surface pCO2, I am wondering how the models handle the possible changes in future biological process in both spatial variation and seasonal magnitudes. Several recent studies have examined temporal and spatial variations in Arctic primary production, as well as the timing of phytoplankton bloom in recent decades in the Arctic Ocean (Song et al., 2021; Lewis et al., 2020; Ardyna et al., 2014). Although it is uncertain that the future changes in biological process will follow the pattern and mechanism which were found in these studies, it is worthy and helpful to have a discussion on how the possible change in the future biological processes may*


nature portfolio

*alter the future pCO2 anomaly. For example, the fall bloom on the Arctic shelve may damper the summer high pCO2 anomaly. It may also weaken the finding in this study that "The tendency for pCO2 to reverse sign in summer during this century is particularly pronounced in the Arctic shelf seas (Fig. 4)."*

We have addressed these issues in the new paragraph quoted at the end of our answer to the previous question. The satellite-based estimates of NPP are extremely valuable despite their large uncertainties. Unfortunately there is no way to know if increasing observed trends in NPP in the Arctic will continue or may actually decline, as suggested by declines in nutrient concentrations (from increased stratification) over the last 30 years in the western Arctic (Zhuang et al., 2022). The 3 references mentioned by Referee #1 are cited in that paragraph.

*Reference:*

*Song, H., Ji, R., Jin, M., Li, Y., Feng, Z., Varpe, Ø., & Davis, C. S. (2021). Strong and regionally distinct links between ice□retreat timing and phytoplankton production in the Arctic Ocean. Limnology and Oceanography, 66(6), 2498-2508.*

*Lewis, K. M., Van Dijken, G. L., & Arrigo, K. R. (2020). Changes in phytoplankton concentration now drive increased Arctic Ocean primary production. Science, 369(6500), 198-202.*

*Ardyna, M., Babin, M., Gosselin, M., Devred, E., Rainville, L., & Tremblay, J. É. (2014). Recent Arctic Ocean sea ice loss triggers novel fall phytoplankton blooms. Geophysical Research Letters, 41(17), 6207-6212.*

*Negative pCO2 anomaly in winter under SSP5-8.5 (CMIP6)*
*Based on current observations, winter sea surface pCO2 is usually close to the atmospheric CO2, even higher in some location due to sea ice formation and possible respiration beneath the ice. I am surprised to see that mean pCO2 anomaly under SSP5-8.5 scenario (CMIP6) for 2091-2100 becomes negative in winter and spring (Extended data Fig. 1b), which is not consistent with the expectation that pCO2 in winter should be above the annual mean or at least close to the annual mean. Among all scenarios in Extended data Fig. 2, the case under SSP5-8.5 (CMIP6) is the only one that mean pCO2 anomaly become negative in winter. Why? Needs more explanation. If it is confirmed to be the case, this result may have an important implication that the Arctic Ocean has a tendency to transform to from neutral state in modern to a weak CO2 sink in the future winter.*

Negative $pCO_2$ anomalies in winter can occur when summer $pCO_2$ is much greater than winter $pCO_2$. Although the present-day winter anomaly of $pCO_2$ is positive and the summer $pCO_2$ anomaly is negative, both of these anomalies are relative to the annual mean ocean $pCO_2$ (not atmospheric $pCO_2$) and adjust as the summer anomaly becomes more positive. The process becomes clear with the 2 new panels added to Fig. 3 (c,d). As summer temperatures grow relative to the annual mean temperature and the summer $pCO_2$ anomaly becomes more positive, the relative neutral line for the annual mean $pCO_2$ essentially moves upward even to the point that it can become more positive than the winter mean $pCO_2$. Furthermore, it is not only in SSP5-8.5 where we find negative winter $pCO_2$ anomalies. They are found in most models in 2091-2100 under SSP5-8.5, in many models under SSP2-4.5, and even in a few models under SSP1-2.6 (Extended Data Fig. 2).

Arctic Ocean $CO_2$ uptake is certainly another key concern and its future change is under debate. We felt it was better to focus here entirely on the impacts on ocean chemistry and biology (acidification) and after this work is published, hopefully take on the additional analysis and write a separate manuscript on the topic of Arctic $CO_2$ uptake.

*Minor comments:*
*Line 67-70: Recent compilation studies of sea surface pCO2 have already showed that summer high pCO2 appeared (splitting from low summer pCO2) in the southern Canada Basin and the Beaufort Sea in the recent decades (DeGrandpre et al., 2020; Ouyang et al., 2020), which was associated with earlier ice*



nature portfolio

*retreat and greater local warming and limited biological CO2 drawdown.*

The text referred to by Referee #1 has been modified to include the following statement:

*Observed summer $pCO_2$ is higher in low-ice years[19], has grown over recent decades as sea-ice retreats[20], and is particularly high under exceptional warming[21-23].*

The first two citations in this text refer to DeGrandpre et al. (2020) and Ouyang et al. (2020).

*Reference:*
*DeGrandpre, M., Evans, W., Timmermans, M. L., Krishfield, R., Williams, B., & Steele, M. (2020). Changes in the arctic ocean carbon cycle with diminishing ice cover. Geophysical research letters, 47(12), e2020GL088051.*
*Ouyang, Z., Qi, D., Chen, L., Takahashi, T., Zhong, W., DeGrandpre, M. D., ... & Cai, W. J. (2020). Sea-ice loss amplifies summertime decadal CO2 increase in the western Arctic Ocean. Nature Climate Change, 10(7), 678-684.*

*Line 94: delete one "and" in the sentence*

Done.

*Line 95: It is better to briefly explain what is "equilibrium climate sensitivity (ESC)" and its implication. So, the readers can more easily follow the text flow.*

In this sentence, we have added a brief parenthetical explanation:

*(ECS, the global-mean surface atmospheric temperature change eventually reached after a doubling of atmospheric $CO_2$)*

*Line 97: How to explain the non-thermal component of summer pCO2 anomaly decreases in the shelves?*

The sentence mentioned refers to Fig. 4, which does show that the nonthermal component of the summer $pCO_2$ anomaly becomes slightly less negative in many of the shelf seas (Fig. 4g-i ). This change on the shelves is explained mainly because the $sC_T$ term becomes less negative, an effect that dominates the opposite tendency for the $sA_T$ term. Other contribtions to the combined nonthermal term (salinity and freshwater terms) are small but add to the tendency of the $sC_T$ term on the shelves, thus enhancing $pCO_2'$. We have added the following sentence to the figure caption:

*The nonthermal component can be further decomposed into its various contributions, as discussed later, showing for instance that its reduction on the shelves is mostly from reduced influence of $C_T$.*

*Line 104: Not all model project warming across the Arctic shelf sea (supplementary Fig. 6), e.g., cooling in the Barents Sea in MPI-ESM-LR.*

The sentence has now been changed to

*Models also project enhanced summer warming across the Arctic, with generally more in the shelf seas (Supplementary Fig. 7) where sea ice recedes earlier.*

*Line 106: Does the statement of "after which the net air-to-sea heat flux becomes so low that it fails to warm surface waters" will still hold true in the 2091-2100 as the sea ice will retreat earlier and warming may extend beyond 1Aug?*

Yes, it still holds true in 2091-2100. In all models, the maximum SST is in August and that does not change with time (Fig 3, Extended Data Fig. 4).

*Line 147: Supplementary Table 2?*

We wrote "(Supplementary Table 3)", and the numbers referred to in the sentence around line 147 do appear in that table, not in Supplementary Table 2. Secondly, this table is listed in order, with the two prior Supplementary Tables mentioned on lines 38 and 96. If we are missing the point of this comment, could Referee #1 please clarify?

*Line 171: What is the possible cause or process driving the salinity normalized AT term to become more important during 2091-2100?*

The increased importance of the $sA_T$ term relative to $sC_T$ term has to do with their balance and is not because of changes in magnitude of their sensitivities, which are nearly identical. Rather it occurs in the models because generally the magnitude of the summer anomaly of $sC_T$ during this century declines more than that of $sA_T$. Less negative $sC_T'$ in the future relative to the present could come from enhanced air-to-sea $CO_2$ transfer due to less sea ice (although increasing summer $pCO_2'$ would work in the opposite direction). The relatively more positive summer $sA_T'$ in the future relative to the present is not found in all models but could come from greater dissolution or less formation of $CaCO_3$ during summer. We have added the following sentence:

*The cause appears to be in the changing balance of the corresponding anomalies, with the magnitude of $sC_T'$ declining more than that of $sA_T'$ (Extended Data Fig. 7).*

Details about possible causes for those changes in $sC_T'$ and $sA_T'$ have been left out because we wish to focus on the much larger thermal effect and we have not done the analysis to confirm our suggestions above for the causes in changes of these secondary factors.

*Line 201: ...for that season's mean and 23±8% for its extreme (Supplementary Table 3). According to Supplementary Table 3, "23±8%" should be. Please double check.*

Supplementary Table 3 does not report numbers for changes in seasonal extremes but rather for seasonal means. Our numbers in that table are consistent with those in the text for the seasonal means. In both we list 15 ± 6% for the summer mean. We did not provide a table for the seasonal extremes. No changes have been made.

*Line 474: Delete one "variables"*

Done.

*Line 550: I am confused about the description of equation terms. When you mentioned "the first right-hand side term in equ (3), are you intending to say "the first left-hand side term"?*

No, we did not intend to say "the first left-hand side term". We actually mean "the first right-hand side term (in parentheses) in equation (3)", as written. There is only one left-hand side term (left of the equal sign) in this equation and that is not in parentheses.

More precisely, what we have written is as follows: "The first right-hand side term (in parentheses) in equation (3) characterizes the effect of increasing atmospheric $CO_2$ (without physical climate change), which affects the sensitivities, ..." This statement is correct because the first right-hand side term contains the change in sensitivities ($\Delta\gamma_i$) dotted with the modern driver anomalies ($X'_{i,0}$). So we have not changed that part of the sentence, but later on in the same sentence we have added "(in separate parentheses)" after "the second and third terms", which further emphasizes that each of the three right-hand side terms is in parentheses.

*Figures:*

*Fig.2: ... Extended Data Fig. 1 shows analogous results from CMIP6 (SSP5-8.5). Add a period. Also, need to explain what is the shading.*

In the Fig. 2 caption we have added "‚ while the shaded region is the uncertainty (±1 s.d., n=9)." We have also added a period after the last sentence.

*Fig. 5: The sentence, "agreement (±1σ, 9 models) but is omitted for the two black curves in the top panel, being already shown in Fig. 2.", seems does not work.*
*Also need to explain what is the shading (uncertainty?)*

This sentence now reads "Shading indicates model agreement (±1 s.d, n=9) but is omitted for the two black curves in the top panel, for which it was already shown in Fig. 2."

*Extended Data Fig. 1: Need to explain what is the shading.*

In this figure caption we have added "‚ while the shaded region is the uncertainty (±1 s.d., n=9)."

*Extended Data Fig. 6: Need to explain what is "True" dots in the plots.*

We have added the following sentence:

*Agreement between the simulated variable (black dots, True) and the sum of the components (solid black) indicates the consistency of the deconvolution.*



nature portfolio

**Referee #2**:

*The manuscript presents a pertinent and scientifically interesting interpretation of future projected changes in the seasonality of the Arctic surface ocean carbon cycle over the 21st century with strong emissions. I think this novel work should be of interest to the broad scientific community and attain the standard rehired for Nature, following text re-organization and streamlining as identified below. The methods are valid, the conclusions are robust, and with minor changes the abstract, summary, and conclusions convey the important points effectively.*

*The comments are organized below by major and minor points.*

*Major Points:*

*First and foremost, I believe that the scientific point of timing emphasized in Fig. 3 and Extended Data Fig. 3 is intriguing. In particular for Extended Fig. 3b and 3d the relatively abrupt (over 20-30 years) transition for the month of maximum pCO2 shows that (albeit often at different CO2 levels) most models exhibit a dramatic shift. I would suggest combining Fig. 3 and Extended Data FIg. 3 to emphasize this in the main text, if this can be allowed with space requirements. The details of the responses of the individual models is sufficiently important to warrant this. But it is also important that the authors return to this question in their interpretation at the end of the paper. Why is it that this transition is triggered at disparate atmospheric CO2 levels for the different models? Much of the analysis here focuses on the phasing and anomalies, but it would be valuable for the authors to connect this with the warming and ice retreat signals more directly, and to identify whether or not there are specific thresholds that trigger this rapid transition.*

We like the suggestion to add the 4 panels from Extended Data Fig. 3 directly to Fig. 3 to allow readers to see more quickly the differences and consistencies between models. But we think it is even more important to add two other new panels to that figure to reveal the evolution of the full seasonal cycle, not just the annual high and low as in the submitted manuscript.   We came to this conclusion after reflecting on the request from both Referee #1 and #2 to provide details as to why the abrupt transition of the annual high in $p$$CO_2$ occurs at disparate atmospheric $CO_2$ levels for the different models. This modified text is also consistent with our strategy to present in one paragraph both Fig. 3 and the related messages that can be garnered only from the CMIP model means before moving on to the following paragraph to discuss model differences (Extended Data Fig. 3).

In any case, the text of the first paragraph has been modified to emphasize that the change in the annual maximum is abrupt (in mid paragraph) while the change in summer $p$$CO_2$' occurs gradually. We also now mention model differences ($2^{nd}$ to last sentence), improving the transition to the details offered in the following paragraph:

*While the crossover $CO_2$ level differs by 256 ppm between CMIP5 and CMIP6 means, and by more among models as shown below, it is remarkably consistent across scenarios for a given model.*

As for why the abrupt transition of the annual maximum is triggered at disparate atmospheric $CO_2$ levels in the different models, we have introduced new text in two places:

(1) two new sentences at the end of the paragraph that introduces Fig. 3, namely

*Despite the abrupt transition in annual maximum p$CO_2$' from winter to summer, the increase in summer p$CO_2$' is a gradual evolution (Fig. 3c,d), the range of which seems to contain the different model behaviours seen in 2091–2100 (Fig. 2 and Extended Data Fig. 1).*

(2) a new paragraph at the end of the section



nature portfolio

*Yet warming from sea-ice retreat does not act alone on $pCO_2'$. Generally opposed in sign is the nonthermal contribution, which varies in models, e.g., due to different biogeochemical components. It is the balance of the nonthermal contribution with the gradually increasing thermal contribution that determines when summer $pCO_2'$ becomes positive and when it surpasses the winter maximum, both abrupt transitions. Models differ in $pCO_2'$ because nonthermal as well as thermal contributions differ, as suggested by their large differences in T' and NPP[5] (Fig. 2 and Extended Data Fig. 1). Although modelled NPP varies greatly and is imperfect, e.g., not capturing observation-based estimates of regional differences[22] and recent changes in phenology[23,24], and observations cannot tell us whether NPP will continue to increase[25] or decline[26] during this century, the use of many diverse models gives confidence that they would encompass the real ocean response. Weighing these contributions requires a quantitative framework.*

This new paragraph also responds to a remark from Referee #1 regarding uncertainties in NPP.

*One a related note, on a first reading of the manuscript, I was also perplexed by Fig. 2b, where at first glance it appears that there is wild model uncertainty in the forced changes in the seasonal phasing of pCO2 anomalies. From this figure alone I wasn't satisfied as a reader that calculating a multi-model ensemble was reasonable for this story, but it was more in the details of the Extended Figures that the authors managed to convince me that this was a reasonable choice to evaluate an ensemble mean rather than looking at two different clusters or groupings for the fields shown in Fig. 2b. I would recommend that the authors move Fig. 1 to the Supplementary Materials, since I think it is confusing to present this ensemble mean view without first showing the behavior of the individual underlying models.*

We appreciate this recommendation but struggle with the idea of first presenting a more complicated figure with all models and 4 variables. Our original simple introductory figure focuses on regional differences to narrow the big picture to the Arctic. That purpose would be defeated by putting it in the Supplementary Information, which is designed for specialists.

However, to try to accommodate this concern and orient readers, we have added the following sentence to the caption of Fig. 2:

*Models fall into 3 groups for simulated $pCO_2'$ in 2091–2100 but may share a common evolution pathway (Fig. 3).*

*In characterizing what is special about the Arctic, I believe that in their discussion the authors should also describe (and reference) what is known about the seasonality component of Arctic amplification as a physical climate phenomenon. This would not require more than a few sentences to make a statement about this and then to connect this to the carbon story.*

In the revised manuscript, we now mention surface atmospheric temperature (SAT). In the concluding paragraph, we say

*Thermally driven increases in summer $pCO_2$ are projected here to amplify and their dominance to become more widespread as atmospheric $CO_2$ increases, seasonal sea ice recedes, and sea surface temperature grows much more in summer than winter, the opposite of surface atmospheric temperature[8].*

To avoid confusion, we avoid going into more detail about Arctic amplification, which is defined in terms of SAT changes, not SST changes. The two variables differ greatly in terms of their projected changes in seasonality. While SST grows more in summer than winter (increased seasonal amplitude), SAT grows more in winter than in summer (reduced seasonal amplitude) (Carton et al., 2015, reference 8 above). The added aside on SAT quoted above seems sufficient since it is SST that directly affects ocean $pCO_2$ and $[H^+]$.


nature portfolio

*Last but not least with the main comments on content, I think that the final section on "Biological Impacts" cold be strengthened by* ***up-fronting more specific information about species and ecosystems that are vulnerable.*** *Given that the authors commented in the earlier part of the manuscript not the work of McNeill and Sasse, it would be helpful if they could comment a bit on the* ***relative importance of hypercapnia, acidification, etc. for organism and ecosystem vulnerabilities.*** *The authors do an effective job of relating both chemical and biological mechanisms that could be important, but it would be greatly beneficial if this could be reshaped to be more directly accessible to a broader audience, including but not limited to ecologists.* ***The authors emphasize pCO2 anomalies, but the absolute levels are also interesting and important.***

Referee #2 has raised 4 points to which we respond below:

(1) At the beginning of the Biological impacts section, we have now inserted a new paragraph that specifically details vulnerable species and ecosystems in the Arctic Ocean. Notably, we mention two keystone species, both noncalcifiers, as well as pteropods, a calcifier. We also point out that one of the noncalcifiers, polar cod, is a central component of the Arctic ecosystem.

(2) Regarding environmental hypercapnia, we do not dwell on that word because McNeil and Sasse (2016) defined it is a threshold (ocean $pCO_2$ > 1000 µatm), while acidification is a process (a reduction in pH). To avoid confusion we focus instead on changes in $pCO_2$ and $[H^+]$, which we demonstrate are linearly related even for seasonal amplitude and timing. This tight relationship makes it more difficult to separate their effects in the real ocean.

Manipulative experiments have been used to try to separate the biological impacts of $pCO_2$ from those of $[H^+]$, but methodological differences have led to different conclusions (Vazquez et al., 2022). We prefer to avoid such details, which are unsettled, but we do now refer to that paper at the end of the paragraph on calcification:

*In some calcifiers, high $[CO_2*]$ may also mediate impacts of ocean acidification[40].*

That final sentence comes just after our mention of the debate about what CO2 system variables control calcification itself.

For fish, the manuscript focuses on $CO_2$ and its transfer across gills which has been brought up as a concern with ocean acidification. In the revised manuscript, we also mention acid excretion across gills as a related subject that needs more attention.

(3) As for making the Biological impacts section more accessible to a broader audience, we have done so with the new introductory paragraph mentioned previously. The second and third paragraphs are indeed more geared for specialists but we hope that most scientists could grasp their basic messages. The fourth and final paragraph, on the effect of warming, should be accessible to all.

(4) We agree that the absolute values of Arctic Ocean $pCO_2$ are also important, namely as the main driver of Arctic air-sea CO2 exchange, the future of which is debated. To do justice to the topic, we feel an entire manuscript would be necessary, and we leave that to future work. That strategy allows us to focus here entirely on chemical and biological impacts (acidification).

*More specific points:*

*In the Abstract, the authors mention the retreat of sea ice, but they don't state there that the changes in seasonality have an abruptness to them that is important. I would recommend mentioning this in the Abstract.*

The abstract has been modified. The relevant sentence now says the following:



nature portfolio

*Often the greater increase in summer $pCO_2$, although gradual, abruptly inverses the chronological order of the annual high and low, a phenomenon not previously seen in climate-related variables.*

*In the final sentence of the Abstract, again I wonder if hypercapnia should also be considered in this context.*

We prefer to avoid adding more complexity to the abstract. Hypercapnia and acidification are closely related. A link is made in the transition between the last two sentences without having to mention the term and explain it. That link is elaborated in the text when we discuss the linear relationship between $[H^+]$ and $pCO_2$ (our equation 2), which makes it difficult to distinguish effects of acidification vs. hypercapnia in the real world.

*Line 27: the study of Rodgers et al. (2008) should be referenced with respect to forced increases in pCO2 seasonality for the surface ocean.*

We have modified the sentence as follows::

*Theory suggests that seasonal variations in ocean pH and CO2 partial pressure ($pCO_2$) should increase[10] as more $CO_2$ invades the ocean and its buffer capacity is reduced, consistent with earlier model and mesocosm studies[11,12].*

Reference 11 refers to Rodgers et al. (2008).

*Line 51: the authors might consider saying "newly revised" rather than "new", unless there is something entirely new about the treatment of the Arctic, in which case this should be explicitly stated.*

We have changed "new" to "recent" and at the end of the sentence have added, "the first to include the non-coastal Arctic Ocean."

*Lines 58-62: See my comments above, I think that Fig. 1 should be moved to the Supplementary Materials.*

We addressed this concern above in our response to the second major point raised by this Referee.

*Lines 64-68: are the authors sure that both CO2 and NPP aren't responding to the warming signal, insofar as sea ice retreat can impact light levels in the Arctic.*

The sentence was ambiguous. It has been split in two, and the second one now reads, "Both appear tied to the earlier retreat of seasonal sea-ice cover."

*Line 160: I think that the section header "Term separation" is too technical, why not change this to be "Mechanistic Attribution" or "Underlying Mechanisms"?*

We have changed this section header to "Quantifying drivers".

*Line 166: should be "salinity-normalized"*

We have added the hyphen.

*Line 222: As noted above, I think it would be much better to begin here with something about specific organisms/ecosystems that are vulnerable, and the relative importance of pCO2 directly acting as a stressor relative to other stressors (acidification and heating). The science here is fine, but the authors should really make this more accessible.*

As mentioned above, we have followed the advice of Referee #2 and added a new paragraph to make this

section more accessible.

*Fig. 2 caption: Need to explain grey shading*

In the Fig. 2 caption we have added ", while the shaded region is the uncertainty (±1 s.d., n=9)."

*Fig. 3 caption: should state why no "minimum" is shown for NPP*

We have added the following sentence:

*The low for NPP is indistinct (Fig. 2) and not shown.*

*Extended Data Fig. 3: should also mention for this figure that small sold circles mark the end of decades*

Done.

*Line 368 and line 372: would it make sense to use the nomenclature BGC and RAD to refer to the esmFixClim1 and esmFdbkl experiments?*

These experiments are not mentioned in the main text but only in the Methods and Supplementary Information. Thus we prefer to remain with their CMIP names and not introduce new acronyms.

**Referee #3**:

The main message of the paper is that the combined effects of ocean acidification and climate change driven warming reverses the seasonal cycle of pCO2 and H+ in the Arctic, turning the summer low of pCO2 and H+ into a seasonal high. The authors use a suite of CMIP5 and 6 models, and a neural network data product, combined with Taylor expansions and a series of specific simulations to determine the effects of different drivers on the inorganic carbon system.

Overall I think this is a well-written, novel, timely paper and I really enjoyed reading it. Figures are clear and support the major findings. I have a few comments that are hopefully useful to the authors.

Title:
I usually do not comment on the title of a paper, but since I am very excited about this work I will give my two cents anyway:
I find that the title is not very clear and is understating what was found in this study. Currently, I don't think the title is accessible to the broad Nature readership.
How about: "The combined effects of OA and climate change shift annual high of pCO2 from winter to summer." Or something along these lines?

The title has been changed to

*Annual high of Arctic Ocean p$CO_2$ could shift from winter to summer*

We've tried to capture the spirit of the suggested title while remaining within *Nature's* 75-character limit. To us, it does appear clearer and less of an understatement.

*Main Text:*
*Again, to make it more accessible to the broader audience I would suggest adding a few sentences about the biological relevance of this finding right on page two, within the first paragraph.*

In the first paragraph of the revised manuscript, we were unable to fit in a few sentences on biological impacts. However, we do now briefly mention mesocosm studies. Furthermore we have a added a new introductory paragraph on this subject at the beginning of the Biological impacts section. Marine organisms are also mentioned in the last sentence of the abstract, just before this paragraph.

*p. 3. L 54-57*
*Sentence is hard to read. Split into two.*

The sentence has been split in two. We've replaced the last part ", thus supporting use of these models for future projections." with a new sentence "This consistency improves confidence in the future projections."

*p. 3, L 58-60*
*This sentence is hard to read. Maybe get rid of "For the future" and exchange end of the century with "end of 2100". Or split up sentence.*

We have now removed "For the future" and replaced "by the end of the century" with "by 2100".

*p. 4 L64 -65*
*I don't see an earlier peak in NPP*

It is subtle, but the earlier peak in NPP is detectable in many models (compare Fig. 2e to 2f and Extended Data Fig. 1e to 1f). It is easiest to see in Extended Data Fig. 4b. No changes were made.

*p. 6 L113*
*"For a wider view" needs to be reworded. It is unclear what the authors are trying to say here.*

The sentence has been reworded. It now reads,

*A second framework was used to assess contributions in the non-idealized experiments made with all the models.*

*P 12 L 222-223*
*Maybe simplify to "CaCO3 dissolution and production both depend on CO32"*
This suggested simplification would change our intended meaning. However, to improve clarity, we changed the sentence to

*In marine calcifiers, dissolution of $CaCO_3$ depends on $[CO_3^{2-}]$, while $CaCO_3$ production may be only correlated with that variable.*

*P 13 L257:*
*This is a bit confusing. As it reads now it says to me: summer pCO2 was already extreme in the past, but in the future it will be 4 times as extreme." But the extreme used to happen in winter... Maybe rephrase?*

For clarity, we have modified this sentence, the text in Supplementary Table 3, and the text where that Table is mentioned in the main text. The sentence that is referred to has now been changed to the following:

*The timing changes projected for ocean $pCO_2$ and $[H^+]$ cause their summer extremes to transition from annual lows to annual highs, enhancing their changes during this century by about one-fourth.*

*P 13 L258-261*
*This is a very important message. Can you make this stronger? Maybe even use the term multistressor, and a bit more info about current research on the impact of these two combined stressors? or even bring in some discussion on compound event?*

Thank you for emphasizing this critical message. As the final sentence of the main text, we would like it to be easily accessible to the general Nature audience. While the suggested terms “multistressor” and “compound event” may resonate with specialists working on ocean acidification, it is our opinion that they are unfamiliar to many and may be perceived as jargon. We think our simpler wording offers a better chance to get the message across to a wider audience. As for research on how thermal vulnerability is affected by ocean acidification, that is mentioned in the previous paragraph of the manuscript and six citations are given. Therefore we hesitate to alter this final sentence, although we remain open.

*P55 L476-478*
*This is confusing. Why CMIP5 CO2 system variable?*

We have deleted the “CMIP5”. The sentence now reads as follows:

*For CMIP6, the only $CO_2$ system variable analyzed was surface ocean $pCO_2$, which was provided by each model group.*

*P57 L 506-507*
*If you debias max temperature doesn't it affect your resutls from the Taylor expansion?*

It certainly would. However, we did not debias temperature or any other variables in our Taylor expansions. This first sentence clarifies this point by adding the word “only”. It now reads:

*Models were debiased only to compute the maximum summer temperature in the shelf seas for 2091--2100 under RCP8.5.*

This point is reemphasized by the last sentence of the paragraph, which reads,

*For other analyses, models were not debiased.*

*Section on Freshwater Taylor series expansion:*
*It is not clear if the authors used a non-zero TA and DIC end member to salinity normalize these variables. If not I would encourage them to look into it. It makes a big difference for the Arctic. See Friis et al., 2003 GEOPHYSICAL RESEARCH LETTERS, VOL. 30, NO. 2, 1085, doi:10.1029/2002GL015898, 2003. If zero TA and DIC endmembers are used, it needs to be shown why it is ok.*

Referee #3 is quite rightly concerned about this issue. How one normalizes with salinity is a key technical point for any freshwater Taylor expansion. Yet studies that report on freshwater Taylor expansions often seem to ignore the concern with salinity normalization raised by Friis et al. (2003). In our study, it would have also been wrong to normalize with a unique salinity such as 35 or a basin-wide average.

Fortunately, our focus is on seasonal anomalies to the annual mean. Thus we were able to normalize locally, basing the freshwater corrections on the ratio of the monthly salinity to the annual mean salinity at each grid cell. This normalization to an annual mean map allows us to avoid the major problems with salinity normalization that were identified by Friis et al. This point is mentioned in the submitted manuscript in the first paragraph of the section "Freshwater Taylor series expansion" of the Supplement, when referring to equation (4) and that has been modified to the following:

*In our case, $S_0$ is the annual mean salinity* ***in each grid*** *cell because the focus is on monthly anomalies relative to the annual mean, a choice also adopted previously[17] that should minimize known problems with salinity normalization[18] .*

Superscipt 17 refers to Landschützer et al. (2018), while superscript 18 refers to Friis et al. (2003). An attempt to clarify this point has been made in the revised manuscript by adding the words "in each grid cell" to that sentence (marked in bold above) and also citing Landschützer et al. (2018), who took the same approach.

*Figure 2*
*Please define shaded area*

In the Fig. 2 caption we have added ", while the shaded region is the uncertainty (±1 s.d., n=9)."

*Figure 3*
*Please define back dashed line*

In the Fig. 3 caption, we have added: "Solid curves are for the annual high; dashed curves are for the annual low."

*Overall comment regarding the use of CMIP5 and CMIP6: It might be useful to mention why simulations from both intercomparison projects were used. What do we learn from one vs. the other?*

We have added the following text to the first paragraph of the Earth system models section of the Methods.

*Using both sets of models improves statistical robustness and takes advantage of improvements in the community of models over the last decade[3] while providing a test to check if conclusions hold across model generations. One improvement in CMIP6 that could be important for the Arctic is that some models have much finer lateral resolution.*

Reference 3 refers to Seferian et al. (2020):

Séférian, R., Berthet, S., Yool, A., Palmieri, J., Bopp, L., Tagliabue, A., ... & Yamamoto, A. (2020). Tracking improvement in simulated marine biogeochemistry between CMIP5 and CMIP6. *Current Climate Change Reports*, *6*(3), 95-119, https://link.springer.com/article/10.1007/s40641-020-00160-0

Another reason to include the CMIP5 models, and not focus only on CMIP6, is to provide a bridge to Kwiatkowski and Orr (2018, Nature Climate Change) who used the same 9 CMIP5 models to quantify the amplification of the seasonal amplitude of $CO_2$ system variables, but who did not discuss simultaneous changes in seasonal timing. Our manuscript reveals that as a group the CMIP6 models were more susceptible to seasonal timing changes than the CMIP5 models. In the text we mention that this greater sensitivity appears to be correlated with the generally greater equilibrium climate sensitivity (ECS) of the CMIP6 models as well as their higher atmospheric $CO_2$ levels, e.g., when comparing SSP5-8.5 to RCP8.5 forcing scenarios.

*Reviewed by Claudine Hauri*

Many thanks to Claudine Hauri and the two other Referees for the quality of their extensive reviews. Their remarks were always constructive and have resulted in major improvements.

**Reviewer Reports on the First Revision:**

Referees' comments:

Referee #1 (Remarks to the Author):

I congratulate the authors for doing a great job in revising the manuscript. I would like to thank the authors for adding Fig 3 c and d, Supplementary Figs.4 and 5, which clearly show the evolution of the full seasonal cycle between the 2000s and 2090s. These new figures provide important and valuable information on revealing that the change in summer pCO2' high is gradual instead of an abrupt change, although the pCO2' seasonal cycle varies among models. The revised manuscript has addressed most of my concerns and I recommend it for publication in its present form.

The only minor comment I have here:
In Fig 3 c and d, Supplementary Figs.4 and 5, the authors used different colors and line types to indicate the pCO2' changes in three stages. That is great. For "(3) annual maximum in summer stage (orange, thick in first decade reaching this stage)", what is the difference between the thick dashed orange line in Fig. 3c and the solid orange line in Fig. 3d? The same question goes for Supplementary Figs.4 and 5. If there is any specific reason, please clarify it.

Reviewed by Zhangxian Ouyang

Referee #2 (Remarks to the Author):

In my opinion, the authors have satisfied the points raised in the first round of reviews.

**Author Rebuttals to First Revision:**

**Referee #1**:

*I congratulate the authors for doing a great job in revising the manuscript. I would like to thank the authors for adding Fig 3 c and d, Supplementary Figs.4 and 5, which clearly show the evolution of the full seasonal cycle between the 2000s and 2090s. These new figures provide important and valuable information on revealing that the change in summer pCO2' high is gradual instead of an abrupt change, although the pCO2' seasonal cycle varies among models. The revised manuscript has addressed most of my concerns and I recommend it for publication in its present form.*

*The only minor comment I have here:*
*In Fig 3 c and d, Supplementary Figs.4 and 5, the authors used different colors and line types to indicate the pCO2' changes in three stages. That is great. For "(3) annual maximum in summer stage (orange, thick in first decade reaching this stage)", what is the difference between the thick dashed orange line in Fig. 3c and the solid orange line in Fig. 3d? The same question goes for Supplementary Figs.4 and 5. If there is any specific reason, please clarify it.*

*Reviewed by Zhangxian Ouyang*

In these figures, the thick orange line has different line patterns because the decade when the annual maximum begins to occur in summer (phase 3) is not the same for the CMIP5 and CMIP6 means. Nor is it the same for the individual CMIP models. Different line patterns distinguish different decades. To make this clearer, we have modified the relevant part of the figure legend as follows:

Evolution of the full seasonal cycle (decadal averages) of $pCO_2$' in the **c,** CMIP5 and **d,** CMIP6 means occurs in three stages: (1) no maximum in summer (black), (2) positive secondary maximum in summer (light blue), and (3) annual maximum in summer (orange). **The thick orange line indicates the first decade reaching stage 3, while line patterns distinguish different decades.** This **evolution** pathway is the rule among models ...

The bold text above highlights the new text.

**Referee #2:**

*In my opinion, the authors have satisfied the points raised in the first round of reviews.*

Thank you.

Many thanks to Dr. Ouyang and anonymous Referee #2 for their efforts to assess the revised manuscript as well as for their extensive previous comments. They have resulted in many improvements.
